# The ethanolamine branch of the Kennedy pathway is essential in the bloodstream form of *Trypanosoma brucei*

**DOI:** 10.1111/j.1365-2958.2009.06764.x

**Published:** 2009-06-30

**Authors:** Federica Gibellini, William N Hunter, Terry K Smith

**Affiliations:** 1 Division of Biological Chemistry and Drug Discovery, School of Life Sciences, University of DundeeDundee DD1 5EH, Scotland, UK; 2 Centre for Biomolecular Sciences, The North Haugh, The UniversitySt Andrews KY16 9ST, Scotland, UK

## Abstract

Phosphatidylethanolamine (GPEtn), a major phospholipid component of trypanosome membranes, is synthesized *de novo* from ethanolamine through the Kennedy pathway. Here the composition of the GPEtn molecular species in the bloodstream form of *Trypanosoma brucei* is determined, along with new insights into phospholipid metabolism, by *in vitro* and *in vivo* characterization of a key enzyme of the Kennedy pathway, the cytosolic ethanolamine-phosphate cytidylyltransferase (*Tb*ECT). Gene knockout indicates that *Tb*ECT is essential for growth and survival, thus highlighting the importance of the Kennedy pathway for the pathogenic stage of the African trypanosome. Phosphatiylserine decarboxylation, a potential salvage pathway, does not appear to be active in cultured bloodstream form *T. brucei*, and it is not upregulated even when the Kennedy pathway is disrupted. *In vivo* metabolic labelling and phospholipid composition analysis by ESI-MS/MS of the knockout cells confirmed a significant decrease in GPEtn species, as well as changes in the relative abundance of other phospholipid species. Reduction in GPEtn levels had a profound influence on the morphology of the mutants and it compromised mitochondrial structure and function, as well as glycosylphosphatidylinositol anchor biosynthesis. *Tb*ECT is therefore genetically validated as a potential drug target against the African trypanosome.

## Introducton

The unicellular eukaryote *Trypanosoma brucei* is the cause of sleeping sickness in humans and Nagana in livestock, in Sub-Saharan Africa. Current treatment has limitations and efforts are being directed into the assessment of drug targets and the development of new drugs. Studies on *T. brucei* metabolism can unravel novel and unique aspects of the parasite's biology that are not only of intrinsic interest and often directly applicable to understanding the disease and to assist the development of new therapies (van Hellemond and Tielens, 2006).

One area with potential as a drug target is the *de novo* biosynthesis of phospholipids (Ancelin and Vial, 1986; Hernández-Alcoceba *et al*., 1997; Urbina, 2006; Choubey *et al*., 2007; Jez, 2007). Phospholipids contribute an important structural role to the membrane and their properties determine membrane fluidity and cell surface charge. They are implicated in a wide variety of cellular processes, including cell division and cell signalling (Dowhan, 1997). Ethanolamine derived phospholipids (GPEtn, phosphatidylethanolamine), which include diacylGPEtn, alkyl-acylGPEtn and alkenyl-acylGPEtn (plasmalogen), can increase the tendency of membranes to form non-bilayer structures, thus influencing membrane fusion and trafficking (Dowhan, 1997; Bakovic *et al*., 2007). These lipids affect the folding, stabilization and activity of integral and membrane-bound proteins and in certain cases GPEtn or GPEtn-derived ethanolamine-phosphoglycerol can be attached to protein residues as a post-translational modification (Signorell *et al*., 2008a). GPEtn is also the donor for the ethanolamine-phosphate capping of the glycosylphosphatidylinositol (GPI) anchor that is required for attachment of proteins to the outer leaflet of the cell membrane (Menon and Stevens, 1992; Menon *et al*., 1993; Imhof *et al*., 2000). This is particularly important for the bloodstream form of *T. brucei*, which relies on a dense coat of GPI-anchored variant surface glycoprotein (VSG) to circumvent the attack of the host immune system (Nagamune *et al*., 2000). Disruption of GPEtn biosynthetic pathways in *T. brucei* is likely to severely impair the parasite homeostasis and thus, the constituent enzymes may represent novel targets for chemotherapy.

The two major pathways for the biosynthesis of GPEtn are the CDP-ethanolamine (CDP-Etn) pathway, also called the Kennedy pathway, and the phosphatidylserine (GPSer) decarboxylation pathway. The CDP-Etn pathway consists of three enzymatic steps. Initially, ethanolamine kinase (EK, EC 2.7.1.82) catalyses the ATP-dependent phosphorylation of ethanolamine (Etn), forming ethanolamine-phosphate (Etn-P), and the by-product ADP. In stage two, the CTP:ethanolamine-phosphate cytidylyltransferase (ECT, EC 2.7.7.14), the subject of this study, utilizes Etn-P and CTP to form the high-energy donor CDP-Etn with the release of pyrophosphate. This reaction is considered to be the rate-limiting step of the Kennedy pathway (Sundler and Akesson, 1975). Diacylglycerol: CDP-ethanolamine ethanolamine-phosphotransferase (EPT, EC 2.7.8.1) catalyses the final reaction of the pathway, utilizing CDP-Etn and diacylglycerol or alkyl-acylglycerol to form diacylGPEtn or plasmalogen, respectively, with CMP as by-product.

An alternative route for the synthesis of GPEtn is the decarboxylation of GPSer by a phosphatidylserine decarboxylase (PSD). This pathway is actually the sole route for GPEtn biosynthesis in *Escherichia coli* and the major one in *Saccharomyces cerevisiae*, although in yeast the Kennedy pathway is also active (Dowhan, 1997). In mammalian cells the relative contributions of these pathways to GPEtn formation is cell-type dependent, with GPSer decarboxylation prevailing in BHK21 and CHO cells and the Kennedy pathway prevailing in most mammalian tissues (hamster heart, rat heart, kidney and liver), and cultured glioma cells (Vance, 2008).

Metabolic labelling and pulse-chase experiments provided some evidence that in *T. brucei G*PEtn could be made from either ethanolamine or serine, even though the Kennedy pathway is used for most of the GPEtn biosynthesis (Rifkin *et al*., 1995). This was recently confirmed by RNA interference in the insect form of the parasite (Signorell *et al*., 2008a,b;), but until now, no detailed studies were performed in bloodstream *T. brucei*. We show, for the first time, that the putative *T. brucei* CTP:ethanolamine-phosphate cytidylyltransferase (*TbECT*) gene (Tb11.01.5730) encodes a functional enzyme, and we characterize the enzyme activity and substrate requirements. Formation of a bloodstream form *T. brucei TbECT* conditional double knockout (cKO) allowed us to demonstrate that *TbECT* is essential and under non-permissive conditions the synthesis of GPEtn and GPI-anchors is severely compromised. We also show that GPSer decarboxylation makes a very minor contribution to bulk GPEtn biosynthesis and it cannot compensate for the loss of the Kennedy pathway in the *TbECT* cKO. These findings suggest there may be therapeutic opportunities in targeting the Kennedy pathway.

## Results and discussion

### Contributions of the Kennedy pathway and GPSer decarboxylation pathway to GPEtn biosynthesis in bloodstream form *T. brucei*

**Fig. 1:**
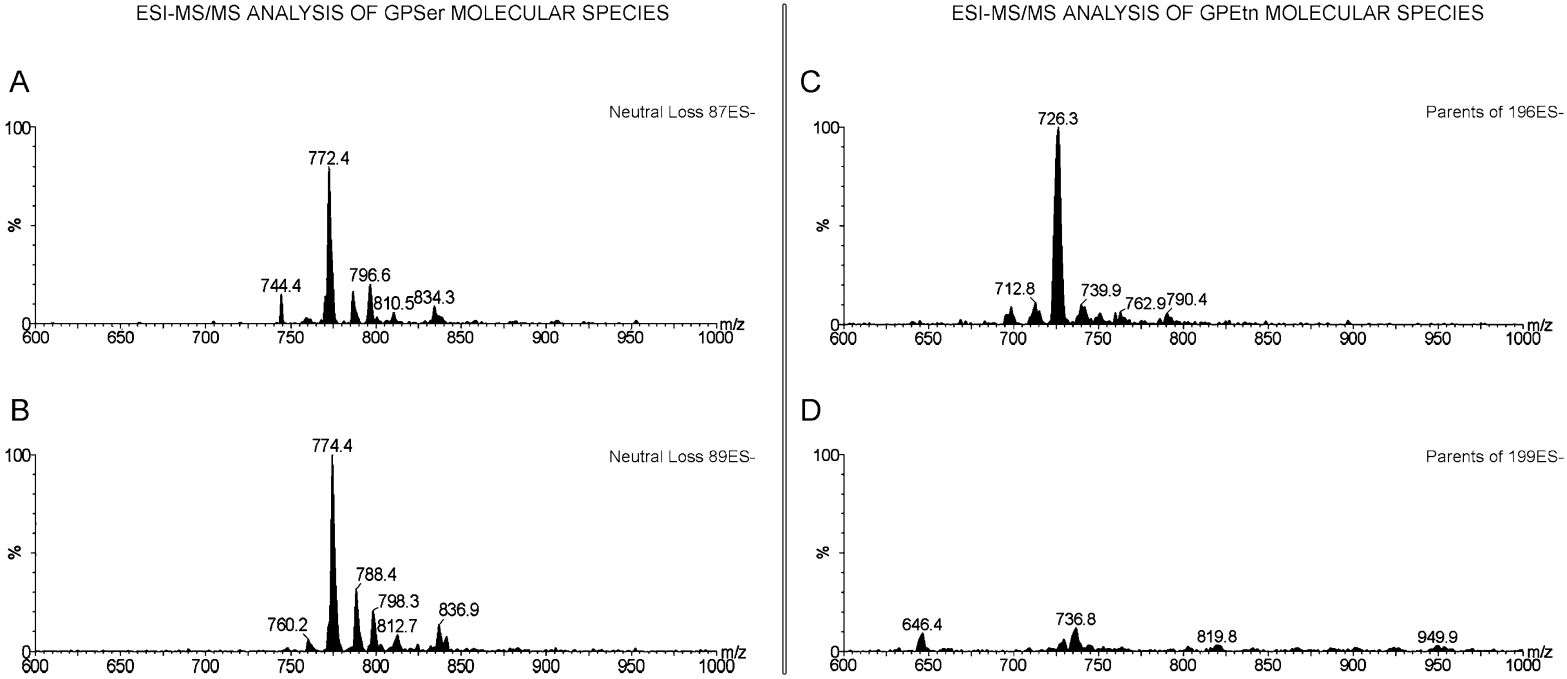
ESI-MS/MS spectra of the following molecular species: GPSer (A); (d_3_)-GPSer (B); GPEtn (C) and (d_3_)-GPEtn (D) in bloodstream *T. brucei* labelled with (d_3_)-serine overnight. Data were normalized to largest peak on display and vertical axes linked in order to directly compare the intensities of non-deuterated versus deuterated phospholipid species.

The relative contribution of the GPSer decarboxylation pathway to GPEtn biosynthesis was assessed by stable isotope labelling with d_3_-serine in normal culturing media overnight, in order to allow adequate time for phospholipid synthesis and a dynamic equilibrium between phospholipids pools to be reached. Subsequent analysis of the phospholipid molecular species by electrospray ionization tandem mass spectrometry (ESI-MS/MS) allowed the overall contribution of the GPSer decarboxylation pathway to GPEtn biosynthesis to be assessed. Upon labelling the d_3_-serine is readily incorporated into newly synthesized d_3_-GPSer, which is virtually super imposable with the pre-existing unlabelled GPSer visualized by neutral loss scans of m/z 89 and 87 respectively (compare Fig. 1A and B).

Similarly, the newly synthesized d_3_-GPEtn formed by GPSer decarboxylation of newly synthesized d_3_-GPSer detected by a parent ion scan analysis for lipids that produce the collison induced 199 m/z fragment in negative ion mode, as opposed to the bulk GPEtn visualized with the collision induced 196 m/z fragment ion (compare Fig. 1C and D). Table S1 shows the annotation of the GPEtn molecular species identified in bloodstream form *T. brucei*.

Although previous work (Rifkin *et al*., 1995) seemed to indicate that bloodstream form *T. brucei* was able to synthesize GPEtn from GPSer via decarboxylation, our experiment clearly shows only trace amounts of d_3_-GPEtn (Fig. 1D), which differ significantly from the *de novo* synthesized GPEtn via the Kennedy pathway (Fig. 1C). This suggests that GPSer decarboxylation contributes little to the biosynthesis of GPEtn under these conditions and it confirms the importance of the Kennedy pathway in the biosynthesis of GPEtn in bloodstream *T. brucei*.

### Identification, cloning and sequencing of *TbECT*

**Fig. 2:**
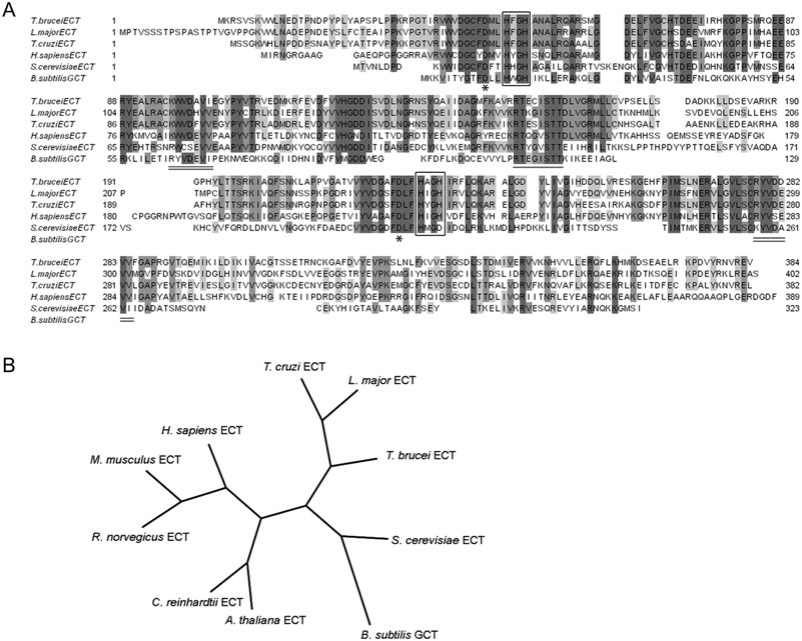
A. ClustalW alignment of the predicted amino acid sequences of *Trypanosoma brucei* ECT (Q382C3-1) with ethanolamine cytidylyltransferases and glycerol-3-phosphate cytidylyltransferases from other eukaryotes: *Leishmania major* (Q4Q5J3), *Trypanosoma cruzi* (Q922E4), *Homo sapiens* (Q99447), *Saccharomyces cerevisiae* (P33412) ECTs and *Bacillus subtilis* GCT. Residues are shaded according to percentage identity using the software Jalview 2.08.1. Boxed and underlined regions are characterisitic signature motif of cytidylyltransferases. B. Phylogenetic analysis of ECTs and GCTs from Fig. 2A plus ECTs from *Mus musculus* (Q922E4), *Rattus norvegicus* (O88637), *Chlamydomonas reinhardtii* (Q84JV7) and *Arabidopsis thaliana* (Q9ZVI9).

The genome sequence of *T. brucei* (Berriman *et al*., 2005) is an invaluable resource for the understanding of the molecular and cellular biology of this important parasite. A putative *TbECT* (Tb11.01.5730) was identified in the *T. brucei* genome database (http://www.genedb.org); the putative open reading frame (ORF) was PCR-amplified from genomic DNA (Lister 427), cloned and the sequence submitted to GenBank Nucleotide Sequence Database with Accession number FM992871. The complete ORF encodes for a protein of 384 residues with a calculated molecular mass of 43.4 kDa. Although *Tb*ECT shows considerable similarity to ECTs from mammalian organisms and shares 40% sequence identity with human ECT, it is unsurprisingly most closely related to putative ECTs from other kinetoplastids such as *Leishmania major* and *T. cruzi*, with 59% and 62% sequence identity respectively (Fig. 2A and B). Like previously characterized ECTs, *Tb*ECT contains two large tandem repeated sequences, probably a result of gene duplication (Nakashima *et al*., 1997; Bladergroen *et al*., 1999). The N-terminal repeat is strongly conserved amongst ECTs and other members of the cytidylyltransferase family: glycerol-3-phosphate cytidylyltransferases (GCT) and the N-terminal domain of choline-phosphate cytidylyltransferases (CCT).

All the signature motifs that characterize the cytidylyltransferase family are present in the *Tb*ECT sequence: two conserved GX(Y/F)DXXHXGH sequences, containing the HXGH motive (^47^HFGH and ^227^HAGH, Fig. 2A, boxed) with the adjacent Asp44 and Asp224 (Fig. 2A, asterisks); one RTX(G/C/S)ISTT motif (^152^RTECISTT) (Fig. 2A, underlined); and the two regions ^96^KWVDAVI and ^278^RYVDDVV (Fig. 2A, double underlined). The structure of *Bacillus subtilis* GCT (Weber *et al*., 1999; Pattridge *et al*., 2003) revealed that the HXGH motif and the RTX(G/C/S)ISTT motif are involved in the formation of the CTP binding site, whereas the third motif is involved in the formation of a dimer interface.

### Recombinant expression and characterization of *TbECT*

To establish if *Tb*ECT is an active ethanolamine-phosphate cytidylyltransferase and to obtain material for the characterization of its properties, *TbECT* was cloned in the expression vector pET20bTEV. This vector encodes for a hexa-histidine tag at the C-terminal of the protein, which can be removed by proteolytic cleavage with Tobacco Etch Virus (TEV) protease (Fig. S1A). The protein was expressed and purified as described in *Experimental procedures*. Typical yields were 20–25 mg per litre of culture. *Tb*ECT could be stored at −80°C in buffer containing 5% glycerol without loss of activity over a period of months.

Matrix-assisted laser desorption/ionization (MALDI) mass spectrum of the cleaved recombinant *Tb*ECT returned a molecular mass of 44 623 Da (theoretical value predicted for recombinant *Tb*ECT: 44 622 Da).

Analytical ultracentrifugation was performed on untagged *Tb*ECT. The sample displayed a single species of mass ∼52 kDa, which agrees with the theoretical mass of the monomer (Fig. S1B).

Interestingly, the rat and human ECTs, with which *Tb*ECT shares high sequence identity, form dimers or higher aggregates in solution (Sundler, 1975; Tie and Bakovic, 2007). The only other reported monomeric ECT is from *S. cerevisiae* (Ohtsuka *et al*., 2006), which shares 34% sequence identity with *Tb*ECT (Fig. 2), despite both of these homologues having the sequence motif predicted to play a role at the dimer interface.

**Fig. 3:**
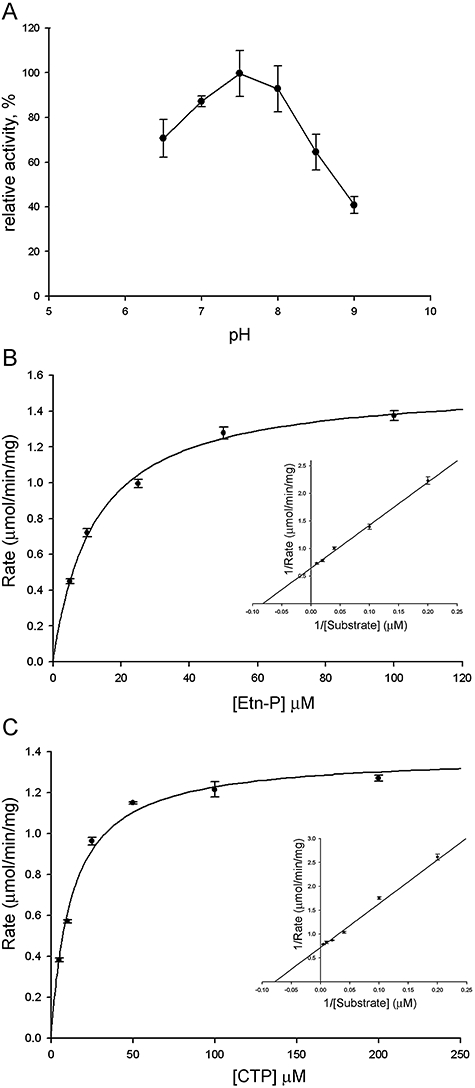
Analysis of CDP-ethanolamine formation by recombinant *Tb*ECT. A. Ethanolamine-phosphate cytidylyltransferase activity was determined by end-point assay as a function of pH as described in *Experimental procedures*. B and C. Determination of *Tb*ECT Michaelis-Menten constants for ethanolamine-phosphate and CTP (inserts show Lineweaver–Burk plots). (B) CTP concentration was held constant (0.1 mM) while ethanolamine-phosphate concentration was varied. (C) Ethanolamine-phosphate concentration was held constant (0.1 mM) while CTP concentration was varied.

*Tb*ECT catalysed the formation of CDP-ethanolamine from ethanolamine-phosphate, in a CTP and magnesium-dependent manner (Fig. S2). At the pH optimum of 7.5 (Fig. 3A) *Tb*ECT displays a *K*m of 12.09 ± 0.87 μM (Fig. 3B) for ethanolamine-phosphate and a *K*m of 12.67 ± 0.78 μM (Fig. 3C) for CTP. These values compare favourably with *K*m values of previously characterized ECTs from rat (*K*m_CTP_ = 53 μM; *K*m_ETN_-*p* = 65 μM (Sundler, 1975) and are about one order of magnitude lower than the corresponding *K*ms in mouse (Tie and Bakovic, 2007). Particularly striking is the unusually low *K*m for the cofactor CTP; this may reflect the relatively low intracellular concentration of this nucleotide triphosphate in *T. brucei* (Hofer *et al*., 1998) compared with the pool present in mammalian cells (Stryer, 1995; Lazarowski and Boucher, 2001) The *V*_max_ of *Tb*ECT is 1.55 ± 0.03 μmol min^−1^ mg^−1^, corresponding to a *kcat* of 1.15 s^−1^.

*Tb*ECT activity was found to be selective for CTP, since no activity was detected with ATP, GTP or UTP (Table S2). The enzyme, however, was able to utilize dCTP, although at a reduced rate, implying that the enzyme is able to discriminate between a deoxyribonucleotide and a ribonucleotide moiety. This is consistent with trace amounts of dCDP-ethanolamine being found in bloodstream form *T. brucei* (Rifkin *et al*., 1995).

Interestingly, *Tb*ECT was unable to accept 2-aminoethyl phosphonate (AEP) as a substrate. AEP is an essential metabolite for the formation of phosphonolipids, which carry a covalent bond between the phosphorous and the carbon of the nitrogenous base (Baer and Stanacev, 1964). However, previous studies (Ferguson *et al*., 1982) failed to detect any phosphonolipids in *T. brucei*, unlike the closely related trypanosomatid, *T.cruzi*, this is reflected both in the absence of enzymes for the biosynthesis of AEP in *T. brucei* (Sarkar *et al*., 2003) and, as shown here, in the substrate specificity of *Tb*ECT.

### Construction of a *TbECT* conditional null mutant

To assess the importance of *Tb*ECT and therefore of the entire Kennedy pathway for the survival of bloodstream form *T. brucei*, a conditional null knockout was generated and characterized.

**Fig. 4:**
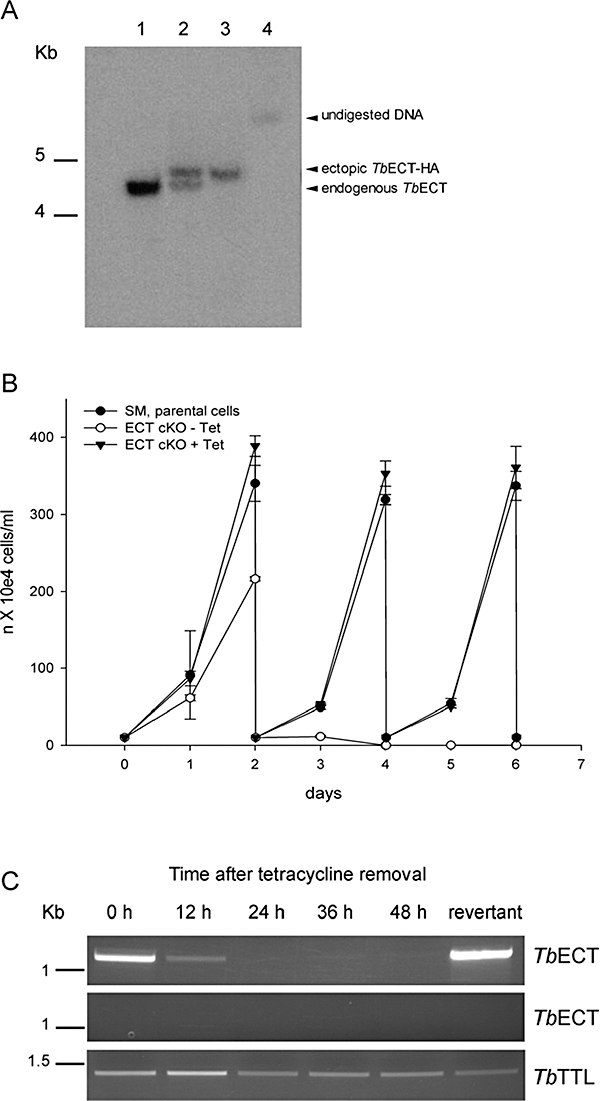
*TbECT* is essential for the survival of bloodstream form *Trypanosoma brucei* in culture. A. Confirmation of genotype of *T. brucei ECT* conditional double knockout cell line. Southern-blot analysis of NcoI-digested genomic DNA (5 μg); the *ECT* ORF probe shows allelic *TbECT* at 4.4 kb and the ectopic copy *TbECT-HATi* at ∼5 kb 1; parental cells (SM) 2; *ΔECT::PAC/ECT-HA*^*Ti*^ 3; *ΔECT::PAC/ΔECT::HYG/ECT-HA*^*Ti*^ 4; Undigested DNA. B. Growth curves of *T. brucei* parental cells (1 – filled circles) and *TbECT* conditional knockout cells grown in the presence (2 – empty circles) or absence (3 – filled triangles) of tetracycline. C. RT-PCR amplification of *TbECT* RNA transcripts from total RNA extracted from *TbECT* conditional null mutants grown in the absence of tetracycline for 0, 12, 24, 36 and 48 h. The upper panel shows RT-PCR products using primers specific for *TbECT*; the middle panel shows a PCR-negative control, without reverse transcriptase; the lower panel shows a loading control using tubulin tyrosine-ligase (*TbTTL*) primers.

Preliminary analysis showed that the *TbECT* gene was present as a single copy per haploid genome and expressed in both bloodstream form and insect life cycle stages (Fig. S3). A conventional mutagenesis approach (Martin and Smith, 2006a) was followed: one allele was replaced by the puromycin resistance gene (*PAC*) generating the Δ*ECT::PAC* cell line; a haemagglutinin (HA)-tagged tetracycline-inducible ectopic copy of the gene was introduced into the ribosomal DNA, yielding the cell line *ECT-HA*^*Ti*^Δ*ECT::PAC*; while maintaining the expression of the ectopic copy by addition of tetracycline, the remaining allele was replaced by a hygromycin resistance gene resulting in the conditional double knockout cell line *ECT-HA*^*Ti*^Δ*ECT::PAC*/Δ*ECT::HYG*. The genotype of the conditional null mutant was verified by Southern blot analysis, where only the ectopic copy is present (Fig. 4A, lane 3).

### *TbECT* is essential to *T. brucei* bloodstream form

To establish whether *ECT* was an essential gene, the cell line *ECT-HA*^*Ti*^Δ*ECT::PAC*/Δ*ECT::HYG* was cultured in the presence or absence of tetracycline and growth rates compared with wild-type. Triplicate cultures for every cell type were inoculated at 1 × 10^5^ cells ml^−1^ and subcultured every two days when necessary. In the presence of tetracycline, that is, in permissive conditions that allow expression of *ECT-HA*^*Ti*^, cells showed normal growth kinetics, comparable to wild-type cells (Fig. 4B). However, when cultured in tetracycline-free media (non-permissive conditions), cells immediately displayed a reduced growth rate and after 48 h they had only attained approximately half the cell density of the wild-type (Fig. 4B). After 72 h an increasing amount of cell debris could be observed and by day 4 cell numbers fell below the limit of detection by light microscopy. RT-PCR confirmed *TbECT* mRNA disappearance after 24 h in non-permissive conditions (Fig. 4C). However, 7–8 days after tetracycline removal live cells were again visible and normal growth rates were resumed. This resumption of growth was due to revertant cells overcoming the tetracycline control, as demonstrated by the reappearance of *TbECT* mRNA in these cells (Fig. 4C). This phenomenon, described many times before in *T. brucei* conditional null mutants for essential genes (Martin and Smith, 2006a), is mostly ascribed to deletion of the tetracycline repressor protein gene (Roper *et al*., 2002).

### *Tb*ECT deprivation affects cellular dimensions and subcellular architecture

Scanning electron micrographs of *TbECT* cKO cells deprived of tetracycline for 36 h suggested a reduction in overall cellular dimensions (almost ‘stumpy-like’) when compared with control cells (Fig. S4A, compare panels 1 and 2 with 3 and 4). Determination of the average cell volume (CASY cell counter, SEDNA Scientific) confirmed an average reduction of up to 30% of cell volume after 48 h of growth in tetracycline free media (Fig. S4B).

**Fig. 5:**
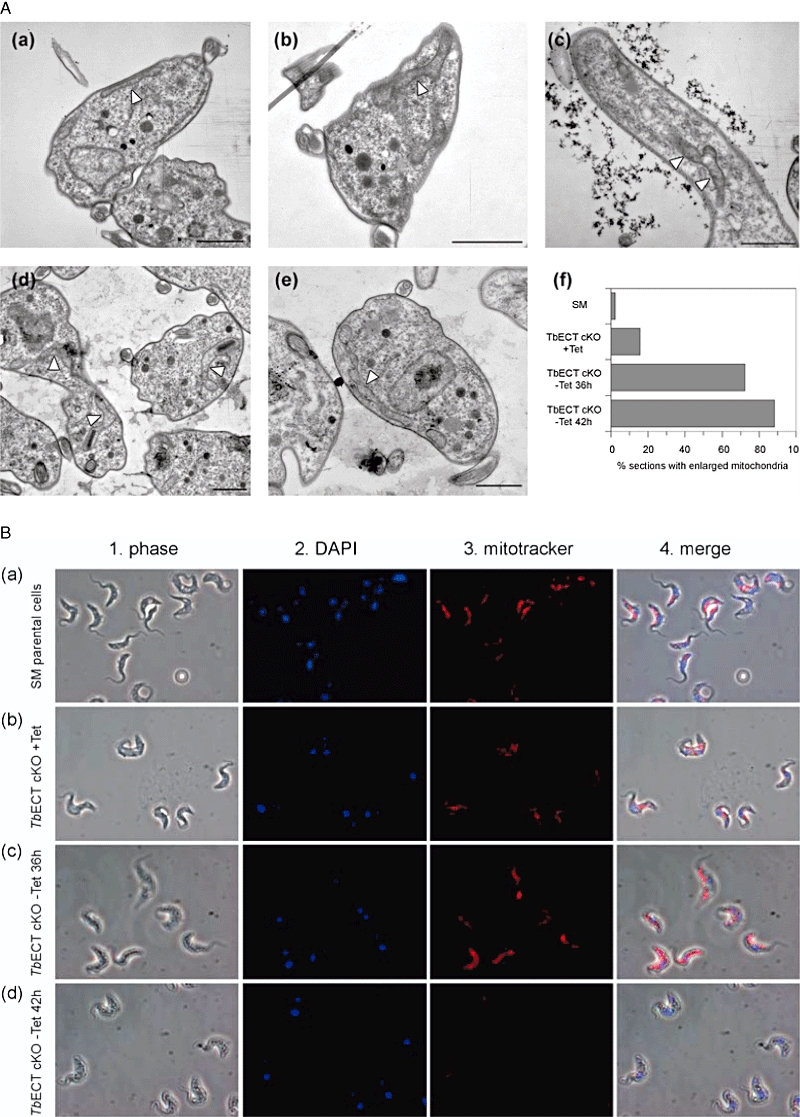
Loss of ECT activity affects mitochondrial morphology and function. A. Transmission electron micrographs of *Tb*ECT cKO cells taken before (a) and after 36 (b) or 42 h (c–e) of tetracycline removal from the media. (f) Quantification of sections displaying an abnormal enlargement of the mitochondria in wild-type and in *Tb*ECT cKO cells at different time points after tetracycline removal (SM parental cell, *n* = 43; *Tb*ECT cKO + Tet, *n* = 83; *Tb*ECT cKO + Tet, *n* = 65; *Tb*ECT cKO -Tet42 h, *n* = 68). Scale bar = 2 μm. B. Mitotracker red CMXRos was used to stain live cells prior to fixation (3); cells were then counterstained in DAPI (2); the corresponding phase contrast images are shown in (1), whereas merged fluorescence-phase images are shown in (4). Mitotracker fluorescence images were collected with the same acquisition time of 542 ms to allow the data to be examined for differences in fluorescence intensity. (a), SM parental cells; (b) *TbECT* cKO cells grown in the presence of tetracycline; *TbECT* cKO cells grown in the absence of tetracycline for 36 h (c) or 42 h (d).

Transmission electron microscopy was used to investigate the subcellular architecture of *TbECT* cKO cells at various time points after tetracycline removal. As shown in Fig. 5A (panels b–e) the main morphological defect appears to be an abnormally enlarged mitochondria, which is usually a thin and narrow tubular structure in the SM parental cells (Fig. 5A, panel a). This abnormal enlargement of the mitochondria was quantified in over 60 suitable transmission electron micrographs at relevant time points. At 36 h without tetracycline ∼70% of the examined mitochondria were significantly enlarged, which increased to ∼90% at 42 h (Fig. 5A, panel f).

To investigate whether this enlargement reflects a loss of function of the organelle, trypanosomes were stained with the membrane potential-dependent fluorescent dye Mitotracker Red (Fig. 5B) and observed by fluorescence microscopy. *TbECT* cKO trypanosomes grown under non-permissive conditions for 36 h still show an accumulation of the dye in the mitochondria, even though the volume of the organelle appears to be increased (Fig. 5B, lane c), suggesting that even if enlarged, the mitochondria is still able to maintain its membrane electrostatic potential. However, after 42 h from tetracycline removal, the dye signal almost completely disappears, suggesting loss of function (Fig. 5B, lane d). Changes in mitochondrial morphology and function have been observed before in mammalian *PSD* mutants (Steenbergen *et al*., 2005) and in *T. brucei* procyclic *ACP* mutants as a result of a decrease of GPEtn in this organelle (Guler *et al*., 2008).

### Phenotype analysis of *TbECT* conditional null mutants by *in vivo* labelling

**Fig. 6:**
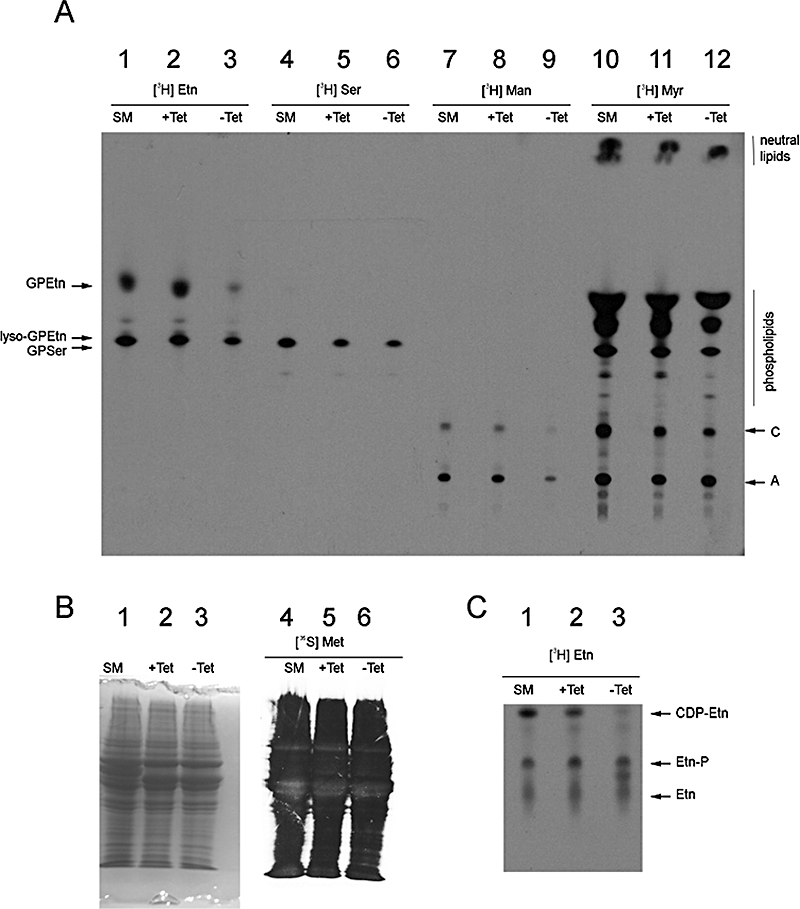
Phenotype analysis of the *TbECT* conditional knockout by *in vivo* labelling. A. SM parental cells (lanes 1, 4, 7 and 10); *TbECT* cKO cells grown in the presence (lanes 2, 5, 8 and 11) or absence of tetracycline for 36 h (lanes 3, 6, 9 and 12) were labelled with either [^3^H]ethanolamine ([^3^H]Etn, lanes 1–3), [^3^H]serine ([^3^H]Ser, lanes 4–6), [^3^H]mannose ([^3^H]Man, lanes 7–9) and [^3^H]myristate ([^3^H]Myr, 10–12), lipids were extracted, desalted and run on HPTLC as described in *Experimental procedures.* Incorporation of radiolabel into lipids was detected by fluorography. Lipid standards of GPEtn, lysoGPEtn and GPSer are indicated on the left. Glycolipids A and C are indicated on the right. B. Cells were labelled with [35S]methionine and proteins extracted and separated on a 4–10% SDS-PAGE gel. Proteins were then detected by Coomassie Blue staining (lanes 1–3) or by fluorography (lanes 4–6). Lanes 1 and 4, SM parental cells; lanes 2 and 5, *TbECT* cKO cells grown in the presence of tetracycline; lanes 3 and 6, *TbECT* cKO cells grown in the absence of tetracycline for 36 h. C. The water-soluble metabolites of the Kennedy pathway were extracted from cells labelled with [^3^H]Etn and run on HPTLC. The metabolites Etn, Etn-P and CDP-Etn are indicated on the right.

The biochemical phenotype of the conditional double knockout cells was investigated by *in vivo* labelling with either [^3^H]Etn, [^3^H]Ser, [^3^H]Man or [^3^H]Myr (Fig. 6) to ascertain the effect of *TbECT* disappearance on GPEtn biosynthesis and potential knock-on effects to GPI anchor biosynthesis and general phospholipid metabolism. The results show a clear decrease of GPEtn formed through the Kennedy pathway in the cKO under non-permissive conditions (Fig. 6A, compare lanes 1–3). This GPEtn decrease does not prompt an upregulation of the GPSer decarboxylation pathway, since no GPEtn is observed from the newly synthesized GPSer (Fig. 6A, lanes 4–6). This represents a fundamental difference with the behaviour of mammalian cells in culture: the Kennedy pathway is the favoured pathway for the biosynthesis of GPEtn when ethanolamine is readily available in the culture media, but the GPSer decarboxylation pathway can compensate under conditions of ethanolamine deprivation (Voelker, 1984).

As expected, blocking the Kennedy pathway at the ECT level results in the disappearance of the CDP-Etn product (Fig. 6C, lane 3). Surprisingly though, this does not translate into an accumulation of the substrate Etn-P (Fig. 6C, lane 3); this could be due to either a downregulation of EK1 or to channelling of the excess of Etn-P into other catabolic or metabolic pathways. This also means that the phenotypical effects of the *ECT* knockout are due to the disappearance of CDP-Etn and the downstream metabolites, i.e. GPEtn, rather than due to an accumulation of toxic levels of Etn-P.

Since GPEtn is the donor of the terminal phospho-ethanolamine group of GPI anchors (Menon *et al*., 1993; Imhof *et al*., 2000), whose biosynthesis has been previously validated as a drug target in the African trypanosome, we tested the possibility that a reduction in cellular GPEtn could produce a knock-on effect on the GPI pathway.

An [^3^H]Man labelling of parental cells and of *Tb*ECT cKO cells grown in permissive conditions highlighted the formation of the expected mature GPI anchor glycolipids A and C (Fig. 6A, lanes 7 and 8) as previously reported (Martin and Smith, 2006b). However, the conditional double knockout cells grown in non-permissive conditions for 36 h displayed a marked decrease in the amount of those labelled species (Fig. 6A, lane 9), meaning that the decrease in GPEtn synthesis, due to deletion of *Tb*ECT, has a detrimental effect on the biosynthesis of mature GPI anchors.

In addition, the [^3^H]Myr labelling shows that there is a slight decrease in the amount of phospholipids synthesized by the conditional double null cells grown in non-permissive conditions for 36 h (Fig. 6A, compare lanes 10–12).

To confirm that the cells were still viable at the time of labelling, their ability of the cells to perform normal rates of protein synthesis was confirmed. The wild-type cells and the conditional double null cells grown in the presence or absence of tetracycline for 36 h showed similar amounts of [^35^S]methionine incorporation into newly synthesized proteins (Fig. 6B), meaning that the biochemical changes detected by the *in vivo* labelling experiments are a consequence of *Tb*ECT deprivation and not due to a general loss of viability. We note that at 48 h, protein synthesis has been partially compromised (data not shown), thus 36 h was chosen for biochemical phenotyping.

### Lipid profile of *TbECT* conditional null mutants by ESI-MS/MS

**Fig. 7:**
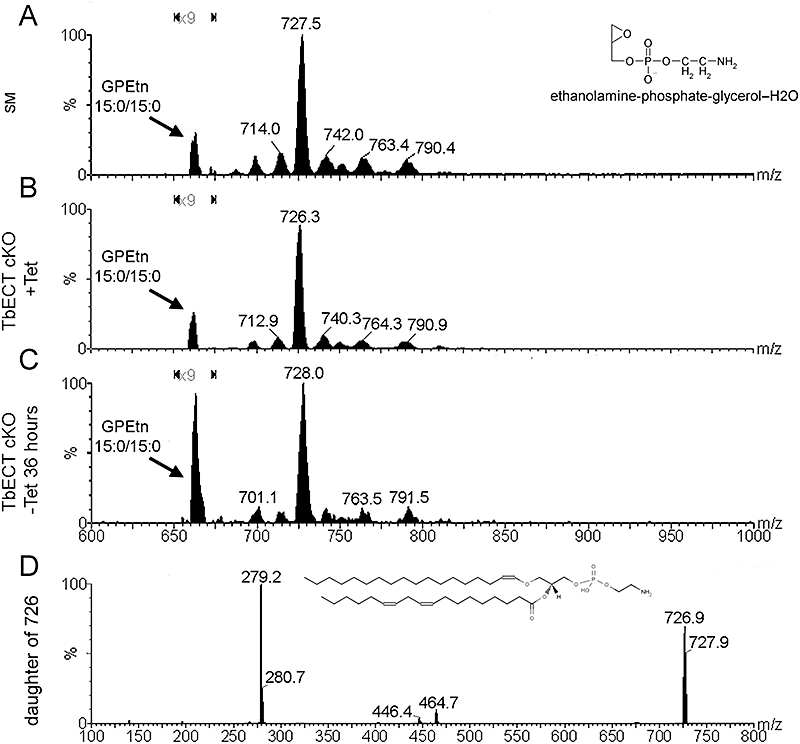
GPEtn phospholipid analysis by ESI-MS/MS. Ethanolamine-containing phospholipids were analysed by ESI-MS/MS in negative ion mode using parent-ion scanning of the collision induced fragment m/z 196. The arrows indicate the peaks of internal standard GPEtn (15:0/15:0), which was subjected to ninefold magnification for clarity. A. SM parental cells. B. *TbECT* cKO cells grown in the presence of tetracycline. C. *TbECT* cKO cells grown in the absence of tetracycline for 36 h. D. Daughter ion spectrum of the m/z 726 [M-H]^-^ ion.

**Fig. 8:**
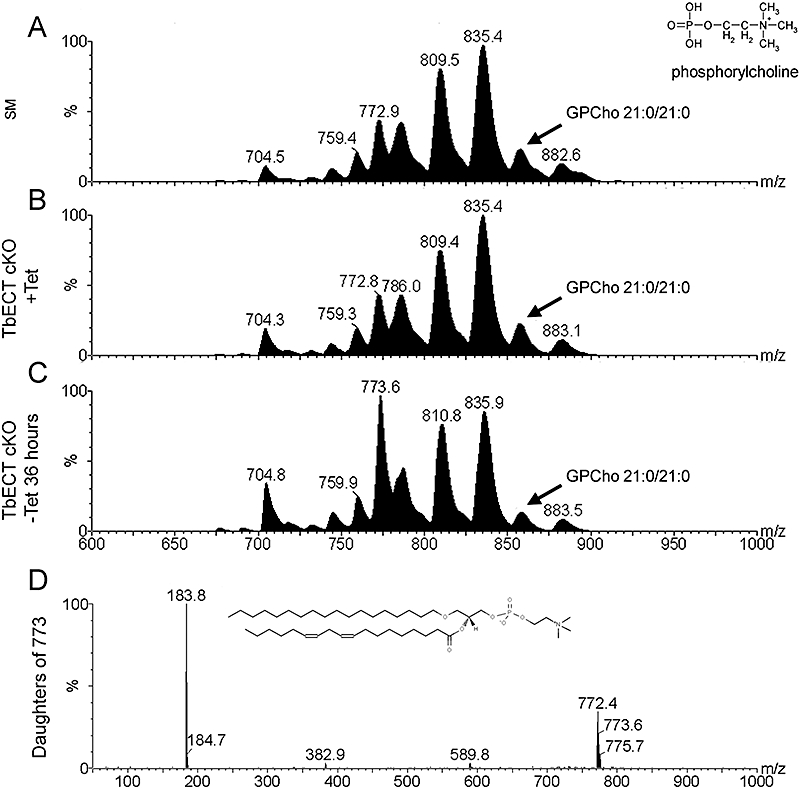
GPCho and sphingomylein phospholipid analysis by ESI-MS/MS. A–C. Choline-containing phospholipids were analysed by ESI-MS/MS in positive ion mode using parent-ion scanning of the collision induced fragment phosphoryl-choline at m/z 184. The arrows indicate the peaks of internal standard GPCho (21:0/21:0). (A) SM parental cells; (B) *TbECT* cKO cells grown in the presence of tetracycline; (C) *TbECT* cKO cells grown in the absence of tetracycline for 36 h. D. Daughter ion spectrum of the m/z 773 [M + H]^+^ ion.

To ascertain if the decrease in GPEtn is due to a decrease in particular GPEtn species or to a global decrease of GPEtn species, and whether this affected the composition of other phospholipid classes, whole-cell extracts from the *Tb*ECT cKO cell line grown in the presence or absence of tetracycline for 36 h were semi-quantitatively analysed by ESI-MS/MS and compared with wild-type (Figs 7 and 8 and Figs S5–8). The negative and positive ion survey scans of the *TbECT* cKO grown under non-permissive conditions contain substantial differences from the control ones, as highlighted by the asterisks (Fig. S5A and B).

A more detailed investigation of the individual phospholipid classes was conducted by parent ion and neutral loss scanning of specific collision induced fragment ions characteristic for each phospholipids class. Initially the effect on GPEtn was investigated. Figure 7 shows that the major GPEtn peak encompassing molecular species in the mass range m/z 724–732 (e-36:3 – a-36:0, Table S1) was considerably reduced in the *TbECT* cKO grown in the absence of tetracycline for 36 h, compared to either the parental cells or the *TbECT* cKO grown in the presence of tetracycline (Fig. 7, compare A and B with C). The principal component of this peak was identified as plasmalogen GPEtn (e-18:0/18:2) by daughter ion scanning of m/z 726 (Fig. 7D). The relative ratio of the intensity of the GPEtn (e-18:0/18:2) peak at m/z 726 with that of the internal standard GPEtn (15:0/15:0), was used to compare the amount of GPEtn in the different samples and showed that the amount of plasmalogen GPEtn after only 36 h of ECT deprivation is reduced to less than a third of the amount contained in the parental cells and the *Tb*ECT cKO grown in the presence of tetracycline.

Parallel to this GPEtn reduction there is a corresponding increase in the phosphatidylcholine (GPCho) peak at m/z 768–776 that includes phospholipids that carry the same kind of fatty acyl constituents (e-36:3 – a-36:0) (Fig. 8, compare A and B with C), with the major species identified by daughter ion scanning as GPCho (a-18:0/18:2) (Fig. 8D). This phenomenon can be attributed to the alkyl-acyl-glycerol not being utilized by EPT in the last step of the Kennedy pathway for lack of CDP-Etn, and thus the excess lipid/substrate is being channelled to the formation of ether GPCho phospholipids through the CDP-choline branch of the Kennedy pathway. Whereas the GPEtn (a-18:0/18:2) species would normally proceed to the formation of the plasmalogen form GPEtn (e-18:0/18:2), the GPCho (a-18:0/18:2) remains in the alkyl-acyl form.

In addition, the cKO cells grown in the absence of tetracycline for 36 h show an increase in both phosphatidic acid (GPA) and phosphatidylglycerol (GPGro) peaks at m/z 749 and 773 respectively (Fig. S6), as assessed by parent ion scanning in negative ion mode of glycerol-cyclic phosphate collision induced ion at m/z 153. These peaks were identified as GPA (18:0/22:5) and GPGro (18:0/18:2) by daughter ion ES/MS-MS (Fig. S6D and E). Since the increase in GPGro (18:0/18:2, m/z 773) is mirrored in the cKO cells grown in the presence of tetracycline, we can hypothesize that the increase in these phospholipid species is a result of the trypanosome metabolism attempting to compensate for a suboptimal amount of GPEtn and/or overall from unused lipid acceptor for GPEtn via EPT.

Interestingly, the total amount and composition profile of both GPSer and phosphatidylinositol (GPIno) are not altered significantly in the cKO in the absence of tetracycline (Figs S7 and S8 respectively). This correlates with the observation that GPSer biosynthesis is only marginally affected by a reduction in GPEtn levels (Fig. 6, lanes 4–6) and seems to exclude the possibility that in bloodstream form *T. brucei* GPSer is synthesized by a base-exchange reaction with GPEtn, a pathway which seems to be present in the procyclic form of the parasite (Signorell *et al*., 2008b).

### *ECT* depletion has a knock-on effect on the GPI anchor biosynthetic pathway

**Fig. 9:**
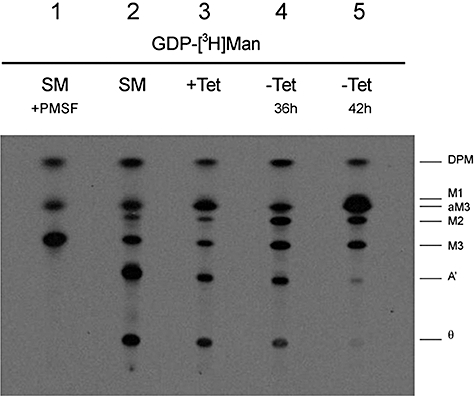
Effects of GPEtn deprivation on GPI intermediates formation. *T. brucei* cell-free system of SM parental cells (lanes 1 and 2), *TbECT* cKO cells grown in the presence (lane 3) or absence of tetracycline for 36 (lane 4) and 42 h (lane 5) were labelled with GDP-[^3^H]mannose in the presence or absence of phenylmethanesulphonyl fluoride, as indicated.

The effect of GPEtn deprivation on the GPI-anchor biosynthetic pathway was studied in more detail by using a trypanosomal cell-free system, an *in vitro* assay in which the throughput through the GPI pathway is maximized (Smith *et al*., 1996). Washed membranes were incubated with GDP-[^3^H]Man and UDP-GlcNAc in the presence of dithiothreitol (DTT) (Fig. 9, lane 2), leading to the formation of dolichol-phosphate-[^3^H]mannose (DPM) and [^3^H]mannosylated GPI intermediates, including Man_1_GlcN-PI (M1), Man_2_GlcN-PI (M2), Man_3_GlcN-PI (M3), Man_3_GlcN-(acyl)-PI(aM3), EtN-*P*-Man_3_GlcN-PI (A′) and the *sn*-2 lyso species of A′ (Θ).

Upon prolonged absence of tetracycline, i.e. 42 h, the formation of the GPI intermediates downstream of the Etn-P addition (glycolipid A′ and Θ) is greatly reduced, and it is paralleled by an increase in the GPI intermediate upstream of the reaction (aM3; Fig. 9, lane 4). This demonstrates that GPEtn deprivation has a very specific effect on the GPI pathway, causing its arrest at the level of Etn-P addition.

As a positive control of a GPI pathway stalled at the level of Etn-P addition, the cell-free system was pre-incubated with phenylmethanesulphonyl fluoride (PMSF), which inhibits the acylation of GPI intermediates (Fig. 9, lane 1), preventing the addition of the Etn-P (Guther *et al*., 1994).

### *Tb*ECT is localized in the cytosol

**Fig. 10:**
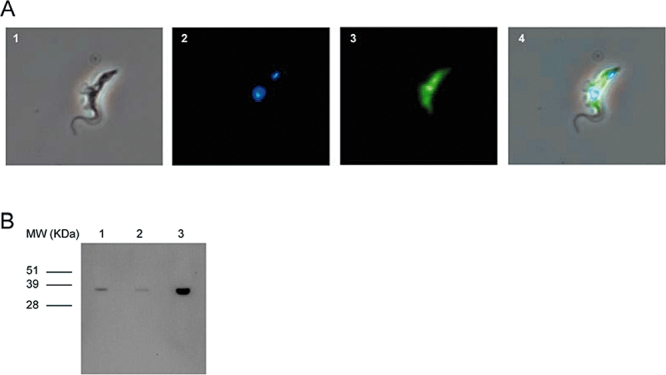
Subcellular localization of ECT-HA in bloodstream form *T. brucei* cells. A. Cells expressing ECT-HA were costained for the HA epitope and for the nuclear marker DAPI. (1) Phase contrast image. (2) DAPI staining. (3) HA epitope staining and FITC detection. (4) Merged image. B. Western blot analysis of subcellular fractions of cells expressing ECT-HA from differential centrifugation. Lane 1, microsomal fraction; lane 2, large granular fraction; lane 3, cytosolic fraction.

Since optimal *Tb*ECT activity occurs at neutral pH (Fig. 4A) and no signal peptides can be identified in the amino acid sequence, a cytosolic localization of the enzyme was hypothesized. The intracellular location was assessed by taking advantage of the HA tag present at the C-terminus of the ectopic copy in the cell line *ECT-HA*^*Ti*^Δ*ECT::PAC*/Δ*ECT::HYG* cultured in the presence of tetracycline. Detection was achieved by immunofluorescence using a primary antibody against the HA-tag and a fluorescein 5′-isothiocyanate (FITC)-conjugated secondary antibody and, as expected, a cytosolic localization was observed (Fig. 10A). This was confirmed by Western blot analysis of subcellular fractions probed with the anti-HA antibody (Fig. 10B).

Cytosolic localization of ECT in *T. brucei* is shared with mammalian cells (Vermeulen *et al*., 1993; Bleijerveld *et al*., 2004); however, it differs considerably from the localization of the enzyme in plants and algae, where studies on *Ricinus communis* endosperm, *Arabidopsis thaliana* and *Chlamydomonas reinhardtii* reveal a mitochondrial (or peri-mitochondrial) and ER localization (Mizoi *et al*., 2006; Wang and Moore, 1991; Yang *et al*., 2004).

### Conclusions

We have here demonstrated that the Kennedy pathway is the sole relevant route for GPEtn biosynthesis in the bloodstream form of *T. brucei* and is required for its survival.

We highlighted differences with the behaviour of mammalian cells in culture in which an alternative pathway, GPSer decarboxylation, can compensate for the loss of the Kennedy pathway due to deprivation of the precursor ethanolamine from the culture media (Voelker, 1984); in bloodstream *T. brucei* the GPSer decarboxylation route appears insignificant in the biosynthesis of GPEtn and is unable to compensate for the loss of the Kennedy pathway when this has been disrupted *via TbECT* gene knockout.

This work also suggested an intriguing difference in the phospholipid metabolism of the two main life cycle stages of the African trypanosome. In the procyclic form GPSer is generated via base-exchange reaction with GPEtn and downregulation of the Kennedy pathway completely blocked GPSer synthesis (Signorell *et al*., 2008a). However, we show that when the Kennedy pathway was disrupted in the bloodstream form of *T. brucei*, GPSer biosynthesis still occurred at levels comparable to the wild-type. Therefore, in this life cycle stage GPSer biosynthesis is more likely to proceed by coupling of serine to CDP-DAG and this represents a major difference in the phospholipid metabolism of the two life cycle stages.

The lethality of the *TbECT* cKO under non-permissive conditions also shows that the corresponding cytidylyltransferase involved in the choline branch of the Kennedy pathway is unable to compensate for the loss of the ethanolamine branch. The generation of a *TbECT* cKO cell line allowed the assessment of the phenotypic changes due to GPEtn deprivation. We showed that the morphology of the mutants and of the mitochondrion was affected at an early stage and later mitochondrial function was disrupted. GPI anchor biosynthesis was also affected and blocked at the level of the Etn-P addition, for which GPEtn is the donor substrate.

Overall, we show that the perturbation in lipid homeostasis has the potential of interfering with numerous cellular processes and structures, which explains the relative rapidity of cell death and makes *Tb*ECT an attractive drug target.

Furthermore, the characterization of the enzymatic properties of ECT provided invaluable information for future experiments aimed at developing inhibitors and new therapeutic agents against the African trypanosome.

## Experimental procedures

### Reagents

Chemicals were purchased from Sigma-Aldrich or Fluka. Restriction endonucleases and DNA modifying enzymes were from New England Biolabs or Promega. [2-^3^H] ethanolamine (50 Ci mmol^−1^) and d-[2-^3^H] mannose (15 Ci mmol^−1^) were purchased from Amersham. [9,10–^3^H(N)]- tetradecanoic acid (myristic acid) (47 Ci mmol^−1^) was from Perkin Elmer, while l-[3–^3^H]-serine (20 Ci mmol^−1^) and GDP-[^3^H]mannose (20 Ci mmol^−1^) were from ARC. l-[^35^S] methionine (1175 Ci mmol^−1^) was from MP Biochemicals. l-[2,2,3–^2^H]-serine (d_3_-Ser) was from CDN Isotopes.

The non-natural phospholipids standards 1,2-diheptadecanoyl-*sn*-glycero-3-phosphate, GPA(17:0/17:0); 1,2-dipentadecanoyl-*sn*-glycerol-3-phosphoethanolamine GPEtn(15:0/15:0); 1,2-dimyristoyl-*sn*-glycero-3-[phospho-rac-(1-glycerol)], GPGro(14:0/14:0); 1,2-diheneicosanoyl-*sn*-glycero-3-phosphocholine, GPCho(21:0/21:0); 1,2-dimyristoyl-*sn*- glycerol-3-[phospho-l-serine], GPSer(14:0/14:0); and 1-dodecanoyl-2-tridecanoyl- *sn*-glycero-3-phospho-(1′-*myo*-inositol), GPI_no_(12:0/13:0) were purchased from Avanti Polar Lipids.

### Cloning and sequencing of *T. brucei* ECT

The putative *TbECT* gene was amplified from *T. brucei* strain 427 genomic DNA together with the 5′-and 3′ untranslated regions (UTRs) of 278 and 402 bp, respectively, using *Pfu* DNA polymerase and the forward and reverse primers 5′-ATAAGTAAgcggccgcGCTAAAGGTGTTGGTGAAACTAGCGC-3′ (F1) and 5′-ATAAGTAAgcggccgcTGGTGAAACAAAACGTTAGTACA-3′ (R2) each containing a NotI restriction site (lower case). The resulting 1.8 kb (ECT and UTRs) fragment was cloned into pCR-Blunt-II TOPO vector (Invitrogen) yielding the pCR-Blunt-II-fl*ECT* construct. Clones were sequenced and compared with the annotated Gene Data Bank sequences.

### *T. brucei* cell culture

Bloodstream form *T. brucei brucei* strain Lister 427, previously genetically modified to express T7 polymerase and the tetracycline repressor protein (Wirtz *et al*., 1999), is referred to here as wild-type. This cell line allows inducible expression of ectopic genes under the control of the T7 promoter and tetracycline operator. Cells were cultured at 37°C and 5% CO_2_ in HMI-9 medium supplemented with 2.5 μg ml^−1^ of G418 to maintain the neomycin drug pressure. *TbECT* conditional null mutant culturing media was supplemented with puromycin, phleomycin, hygromycyn and tetracycline at the concentrations in the section below. Cells were counted daily and the average cell volume recorded with a CASY Cell Counter and Analyser System Model TT using lower and upper cell dimension limits of 2.40 and 5.70 μm.

### Stable isotope labelling of bloodstream form *T. brucei*

2.5 × 10^7^*T. brucei* bloodstream form cells at a density of 0.5 × 10^6^ cells ml^−1^ were incubated overnight at 37°C in HMI-9 media supplemented with 1 mM d_3_-Ser. Total lipids were extracted by the method of Bligh and Dyer (1959), and samples were analysed with a Micromass Quattro Ultima triple quadrupole mass spectrometer equipped with a nano-electrospray source. [M-H] adducts of unlabelled GPSer (d_3_)-labelled GPSer, unlabelled GPEtn and (d_3_)-labelled GPEtn were monitored by neutral loss scanning for m/z 96 and 99 and by parent ion scanning for m/z 196 and 199 respectively. Each spectrum encompasses at least 50 repetitive scans.

### Construction of *T. brucei* ECT conditional gene knockout

To construct the *T. brucei* gene replacement cassettes, the 5′ and 3′ UTRs adjacent to the *TbECT* ORF were amplified from the pCR-Blunt-II-fl*ECT* construct using the primers F1 and 5′-GGATCCGTTTAAACTTACGGACCGTCAAGCTTCAAAGGGGTAAAACCCACAGATAC-3′ (R1) for the 5′-UTR; and the primers 5′-AAGCTTGACGGTCCGTAAGTTTAAACGGATCC ACTTTTATCCTTCGTGAGGAAT-3′ (F2) and R2 for the 3′ UTR. The amplified products were then used in a knitting PCR, that utilized a short linker region (underlined) containing the restriction sites BamHI, HindIII and PmeI in order to anneal together the 5′-to the 3′ UTR.

A HindIII restriction site internal to the sequence of the 3′-UTR was silenced by site directed mutagenesis using the forward primer 5′-CCATCAAAAAAGAAGGAGAAGCCTTGCATGGATAAGTAGAG-3′, the reverse primer 5′-CTCTACTTATCCATGCAAGGCTTCTCCTTCTTTTTTGATGG-3′ and the QuickChange Site Directed Mutagenesis Kit (Stratagene).

The stitched UTRs were then ligated into pGEM-5Zf(+) (Promega) via the NotI sites and the antibiotic resistance markers hygromycin phosphotransferase (HYG) or puromycin acetyltransferase (PAC) were ligated between the BamHI and HindIII restriction sites.

To generate the tetracycline inducible ectopic copy of the *TbECT* gene, the ORF was amplified by PCR from the pCR-Blunt-II-fl*ECT* construct using *Pfu* polymerase and the forward and reverse primers 5′-CCCAAGCTTGGGATGAAACGGTCGGTGTCGAAGGT-3′ and 5′-CCTTAATTAAGGCACCTCTCTGACATTTCTGTACA-3′. The PCR product was then cloned into the vector pLew100, which contained a phleomycin resistance cassette (Wirtz *et al*., 1999), using the HindIII and *Pac*I sites (underlined).

These constructs were purified using a QIAprep Miniprep Plasmid Kit (Qiagen), linearized with NotI, precipitated with sodium acetate/ethanol and dissolved in sterile water to a final concentration of 2 mg ml^−1^. The DNA was then electroporated into *T. brucei* bloodstream form cells: 3 × 10^7^ cells were re-suspended in T-cell Nucleofector solution and transfected using the program X-001 on an AMAXA Biosystems nucleofector. Transfected cells were left to recover in HMI-9 medium overnight or for 6 h; a 10-fold dilution of the cells was also applied at this stage. After recovery, drug selection was achieved in the presence of a final concentration of 0.1 μg ml^−1^ of puromycin for cells containing the PAC gene; 2.5 μg ml^−1^ of phleomycin for cells transfected with the pLew100 construct; and 4 μg ml^−1^ of hygromycyn for cells containing the HYG gene. Before deletion of the second *Tb*ECT allele expression of ectopic *Tb*ECT was ensured by addition of 1 μg ml^−1^ of tetracycline. This concentration was maintained for culturing of the *Tb*ECT conditional null mutant, whereas arrest of ECT gene expression was achieved by washing cells three times in tetracycline-free HMI-9 prior to culturing in tetracycline-free HMI-9.

### RNA isolation and cDNA synthesis

Total RNA was isolated from bloodstream form *T. brucei* using the RNeasy mini kit (Qiagen). *TbECT* specific cDNA was generated and amplified using the specific forward 5′-GAGATATACATATGAAACGGTCGGTGTCGAAGG-3′ and reverse 5′-CCGGATCCTCACACCTCTCTGACATTTCTGTA C-3′ primers using the SuperScript III One step RT-PCR kit with Platinum *Taq* (Invitrogen). As a negative control to exclude DNA contamination of the RNA sample, reverse transcriptase was omitted from the reaction and replaced with GoTaq polymerase (Promega). Tubulin tyrosine-ligase (*TbTTL*) cDNA was also amplified using the forward and reverse primers 5′-GGAATTCCATATGATGAGTGAGTTCCCGTTGGTGTC-3′ and 5′-CGCGGATCCGCGTCACGTGCTCCCGATAGGCAATTC-3′ to show equal RNA input. The PCR products were then run on a 1% agarose gel.

### Southern and Northern blotting

Southern and Northern blotting were performed essentially as described before (Martin and Smith, 2006a), using *TbECT* specific probes generated by PCR using the primers previously described for the amplification of the *ECT* ORF for ligation into pLew100.

### Subcellular localization studies

Immunofluorescence, subcellular fractionation and differential centrifugation were carried out on the *TbECT* cKO cells grown in the presence of tetracycline as described before (Martin and Smith, 2006b).

### Mitotracker staining

Mid-log bloodstream form *T. brucei* parental SM cells and *TbECT* cKO cells grown in the presence or absence of tetracycline for 36 and 42 h were incubated for 10 min at 37°C in HMI-9 medium containing 50 nM Mitotracker Red CMXRos (Molecular Probes). Cells were then harvested, washed in HMI-9 medium and incubated for further 30 min in the absence of Mitotracker. Cells were collected by centrifugation (800 *g*, 10 min), washed in PBS and fixed with 4%(w/v) paraformaldehyde in PBS. Cells were counterstained with 4,6-diamidino-2-phenylindole (DAPI, 2 μg ml^−1^), washed in PBS, let adhere to polylysine slides and the slides mounted.

### *In vivo T. brucei* metabolic labelling

For metabolic labelling 2 × 10^7^ mid-log cells were centrifuged (800 *g*, 10 min) and washed in: serine free Minimal Essential Media (MEM), for [^3^H]serine and [^3^H]ethanolamine labelling; methionine free MEM, for [^35^S]methionine labelling; glucose free MEM, for [^3^H]mannose labelling and MEM supplemented with defatted BSA precoupled with [^3^H]myristate for [^3^H]myristate labelling. The cells were then re-suspended in the appropriate media at the final concentration of 1 × 10^7^ cells ml^−1^. Cells were labelled for 1 h at 37°C with 50 μCi ml^−1^ of the relevant radiolabelled species. Two aliquots containing each 1 × 10^7^ cells were then collected from each labelling experiment by centrifugation (800 *g*, 10 min) and used for lipid and protein analysis.

Lipids were extracted in chloroform:methanol:water (10:10:3) for 1 h, the supernatant removed and the pellet extracted with fresh 10:10:3 for 1 h. The supernatants were pooled and dried under a stream of nitrogen prior to desalting using butanol/water partitioning. Lipids were separated by HPTLC using silica 60 HPTLC plates with chloroform : methanol : water (10:10:3 v/v) as the solvent. The aqueous phases containing the ethanolamine metabolites were dried under a nitrogen stream and separated by HPTLC using silica 60 HPTLC plates in a solvent system composed by 100% ethanol : 0.5% sodium chloride : 25% ammonium hydroxide (10:10:1). Cold ethanolamine, ethanolamine-phosphate and CDP-ethanolamine standards were run in parallel and detected with ninhydrin. Radiolabelled species were detected by fluorography at −80°C, after spraying with En^3^hance™ and using Kodak Biomax MS film with an intensifying screen. Proteins were separated on a 4–12% SDS-PAGE gel and visualized by Coomassie blue staining. Destained gel was soaked in En^3^hance™ (NEN) for 30 min, washed with water twice, soaked in 10% glycerol and dried. The dried gel was then exposed to Kodak Biomax MS film for 7 days at −80°C.

### Electron microscopy

For scanning electron microscopy the samples were fixed directly in HMI-9 media by adding glutaraldehyde to a final concentration of 2.5% (v/v) and processed as described before (Urbaniak *et al*., 2006). Samples were then examined using a Philips XL 30 environmental scanning electron microscope operating at an accelerating voltage of 15 Kv.

For transmission electron microscopy cell pellets were fixed for up to 24 h with Peter's fixative (1.25% glutataldehyde, 1% paraformaldehyde in 0.08 M sodium cacodylate pH 7.2 with 0.02% calcium chloride). Samples were rinsed twice in 0.08 M sodium cacodylate pH 7.2 then postfixed/stained with 1% osmium tetraoxide (aq) for 1 h. Specimens were then rinsed twice in distilled water before fixation/staining in 3% uranyl acetate (aq) for 16–18 h. Samples were rinsed twice in distilled water, dehydrated through a graded ethanol series then washed twice in propylene oxide. Samples were then infiltrated with a 50% (v/v) mixture of Durcopan resin and propylene oxide for 24 h on a rotary mixer at room temperature. After infiltration for a further 24 h in 100% Durcopan resin, samples were polymerized at 60°C for 24 h. Sections were cut using a Leica Ultracut UCT microtome, mounted on pioloform coated copper grids, post stained with 3% uranyl acetate (aq) and Reynold's lead citrate and examined using a JEOL-1200 EX transmission electron microscope operating at a accelerating voltage of 80 Kv.

### Electrospray tandem mass spectrometry

Total lipids from 1 × 10^8^ trypanosomes were extracted by the method of Bligh and Dyer (1959) with un-natural lipid standards added prior to lipid extraction [1 nmol of standard per sample, with the exception of gpino(12:0 13:0) of which only 0.2 nmol were added per sample]. Samples were analysed with a Micromass Quattro Ultima triple quadrupole mass spectrometer equipped with a nano-electrospray source, as described previously (Guler *et al*., 2008). Each spectrum encompasses at least 50 repetitive scans.

### *Tb*ECT recombinant protein expression and purification

The *ECT* ORF was PCR amplified from the pCR-Blunt-II-fl*ECT* construct with *Pfu* polymerase using the forward primer 5′-GGAATTCCATATGATGAAACGGTCGGTGTCGAAG-3′ containing a NdeI restriction site (underlined) and the reverse primer 5′-GGATCCC**CTTGAAAATACAGGTTTTCGCCGCC**GGTACCCACCTCTCTGACATTTCTGTACACATCTGGC-3′, which contains a BamHI restriction site (underlined) and encodes a product carrying a TEV protease cleavage site (bold). The amplicon was purified (QIAquick PCR purification kit, Qiagen), subcloned into pCR-Blunt II TOPO (Invitrogen) and sequenced. Using the NdeI and BamHI restriction sites the putative *TbECT* was ligated into the expression vector pET-20b (Novagen), generating the construct pET20b-*TbECT*-TevP-His_6_, which incorporates a C-terminal TEV cleavable hexa-histidine tag when expressed.

The pET20b-*Tb*ECT-TevP-His_6_ construct was transformed in BL21(DE3) Codon plus RIL cells and clones selected on LB-agar plates containing carbenicillin (100 μg ml^−1^) and chloramphenicol (25 μg ml^−1^). Starter cultures were grown at 37°C from single colonies in 10 ml of LB medium containing carbenicillin (50 μg ml^−1^) and chloramphenicol (12.5 μg ml^−1^), for at least 5 h. The starter culture was then used to inoculate 1 l of autoinduction media (Studier, 2005) and the cells were grown at room temperature for 24 h. Cells were harvested by centrifugation (3500 *g*, 20 min, 4°C) and re-suspended in buffer A (50 mM HEPES pH 7.5, 300 mM NaCl, 10 mM imidazole, 2 mM β-mercaptoethanol and 5% glycerol). Cells were lysed in the presence of Dnase I using a French press and the lysate cleared by centrifugation (35 000 *g*, 30 min, 4°C).

The cleared lysate was applied to a 5 ml HisTrap™ column (GE-Healthcare) preloaded with Ni^2+^. Unbound proteins were removed by washing the column with 15 column volumes of buffer A, while *Tb*ECT was eluted with an imidazole gradient in the same buffer.

Fractions containing *Tb*ECT were pooled and the His-tag was cleaved with TEV protease (1 mg of protease per 10–20 mg of protein substrate) containing an uncleavable His-tag, while dialysing against buffer A. The cleaved *Tb*ECT was purified from the remaining His-tagged version of *Tb*ECT, the tag and the His-tagged TEV protease with a second round of nickel ion affinity chromatography, as described above. Fractions containing cleaved *Tb*ECT were pooled, the purity of the sample was checked by SDS-PAGE, and aliquots were snap frozen in liquid nitrogen and stored at −80°C.

In order to assess the molecular weight and the oligomeric state in solution of *Tb*ECT, the protein was purified as above but glycerol, which can interfere with such analyses, was omitted from the purification buffers. Exact molecular weight was assessed by MALDI time-of-flight mass spectrometry. Sedimentation velocity experiments were performed (wavelength of 280 nm, rotor AN50-TI at 45 000 r.p.m. and 20°C), using a Beckman Coulter XL-1 analytical ultracentrifuge. The samples were run in 25 mM HEPES pH 7.5, 50 mM NaCl, 2 mM DTT, 2 mM MgCl_2_ at concentrations of 0.25, 0.5 and 0.75 mg ml^−1^. Samples were centrifuged simultaneously and A_280_ measurements taken at five-minute intervals for 16 h. The resultant data were analysed using the programs SEDFIT and SEDNTERP (Schuck, 2000; Lebowitz *et al*., 2002).

### Assay of *TbECT* activity

Ethanolamine-phosphate cytidylyltransferase activity was measured by a malachite green colorimetric assay in 96-well plate format using a SpectraMAX 340PC plate reader (Molecular Devices). The assay contained 25 mM Bis-tris propane pH 7.5, 5 mM MgCl_2_, 1.425 mg ml^−1^ of recombinant *Tb*ECT, 0.1 unit of inorganic pyrophosphatase (Sigma) and varying concentrations of CTP and Etn-P, in a total volume of 50 μl. The reaction was carried out for 5 min at room temperature and stopped by the addition of 100 μl of malachite green reagent (Biomol Green), and the absorbance recorded at 620 nm. Controls were conducted to make sure enzyme activity and product formation displayed a linear relationship in the time span of the assay. All enzyme assays were conducted at least in triplicate. Enzyme Kinetics, SigmaPlot (SPSS), was used to analyse and fit the kinetic data. Substrate recognition was investigated by substituting either Etn-P or CTP with substrate analogues or alternative nucleotide phosphates.

The pH optimum of the reaction was assessed by measuring *Tb*ECT activity at various pHs: Bis-Tris methane (pH 6.5, 7.0), Bis-Tris propane (pH 7.5, 8.0, 8.5) or Glycine (pH 9.0).

The production of CDP-ethanolamine by the ethanolamine-phosphate cytidylyltransferase assay was assessed using a modified method of Tijburg *et al*. (1992). Briefly, 2 μg of purified protein are incubated with a reaction mixture (total volume 50 μl) of 100 mM Tris pH 8, 10 mM MgCl_2_, 5 mM CTP, and 4 mM Etn-P at 37°C for 20 min and quenched by boiling at 100°C for 5 min. Substrates and products were separated by HPTLC using silica 60 plates with 100% ethanol : 0.5% sodium chloride : 25% ammonium hydroxide (10:10:1, v/v) as solvent. Substrates and products were visualized by spraying with ninhydrin (0.2% in water-saturated butan-1-ol).

### Cell-free system assay of GPI biosynthesis

Membranes of *T. brucei* wild-type and *TbECT* conditional null mutants grown in the presence or absence of tetracycline for 30, 36 and 42 h were isolated and prepared as described previously (without the addition of tunicamycin prior to lysis) (Smith *et al*., 1997), snap frozen in liquid nitrogen and stored at −80°C until required. The cell-free system assay was carried out and visualized as described before (Smith *et al*., 1997) using 1 × 10^7^ cell equivalents per assay, GDP-[^3^H]Mannose (0.3 μCi per 10^7^ cell equivalents) with or without 1 mM PMSF.

## Note added in proof

The nucleotide sequence reported in this paper has been submitted to the GenBankTM/EBI Data Bank with accession number FM992871.
