# Supplementary material for: The ethanolamine branch of the Kennedy pathway is essential in the bloodstream form of Trypanosoma brucei
Source: Mol Microbiol. 2009 Jun 30;73(5):826–43. doi: 10.1111/j.1365-2958.2009.06764.x (PMC2784872; doi:10.1111/j.1365-2958.2009.06764.x)
Supplement: Supplementary file 1 [file mmi0073-0826-SD1.pdf]

SUPPORTING INFORMATION

Figure S1

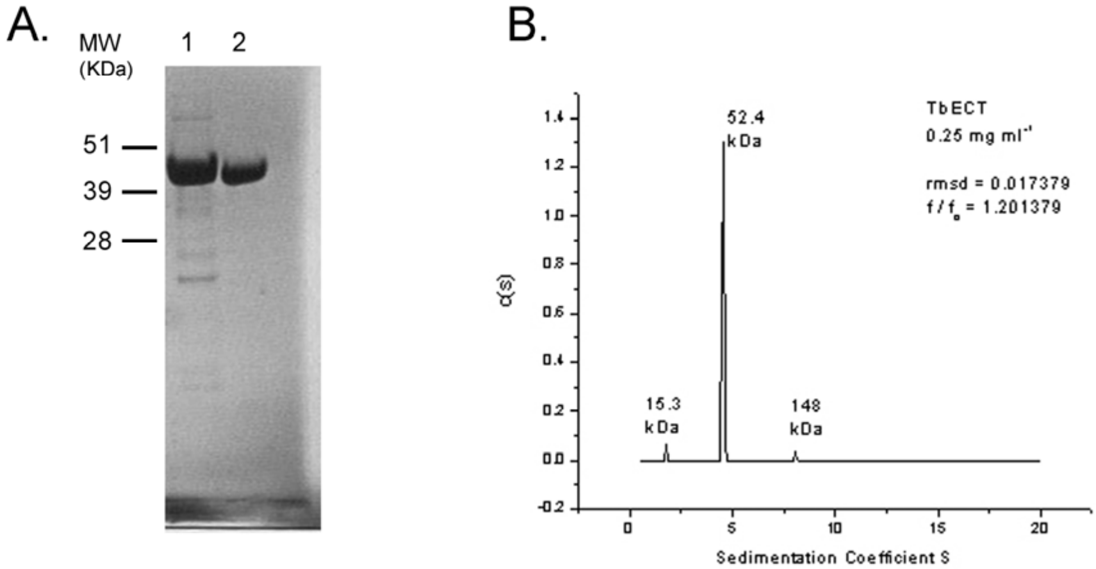

Figure S1. Expression and purification of recombinant *Tb*ECT in *E. coli.*
(A) Protein samples after each purification step were separated on a 10% SDS-PAGE gel and stained with Coomassie Brilliant Blue. Lane1: Protein sample after the first nickel ion affinity chromatography step; Lane 2: Protein sample after tag removal and second nickel ion affinity chromatography step. **(B)** Plot of the distribution of the sedimentation coefficients generated from Analytical Ultracentrifugation (AUC) of *Tb*ECT at 0.25 mg/ml in buffer containing 25 mM Hepes pH 7.5, 50 mM NaCl, 2 mM DTT, 2 mM $MgCl_2$.

Figure S2

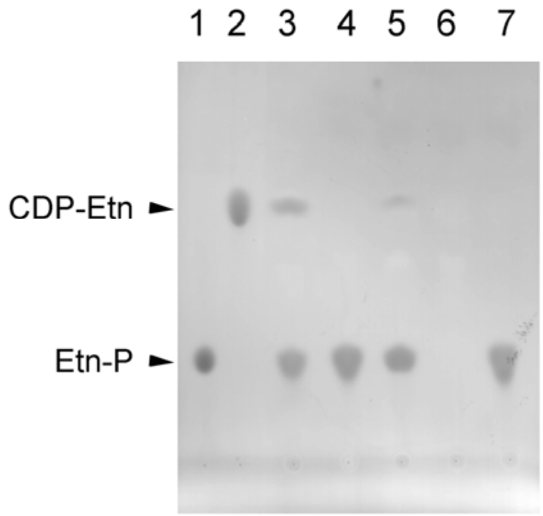

Figure S2. The substrate ethanolamine-phosphate (Etn-P) and the product CDP-ethanolamine (CDP-Etn) of the ECT reaction were separated by HPTLC and detected by ninhydrin. Lane1, Etn-P standard; Lane 2, CDP-Etn standard; Lane 3, with *Tb*ECT; Lane 4, negative control with no enzyme added to the reaction mixture; Lane 5, control with no Mg^{2+} added to the reaction mixture; Lane 6, negative control with no Etn-P added to the reaction; Lane 7, negative control with no CTP added to the reaction.

Figure S3

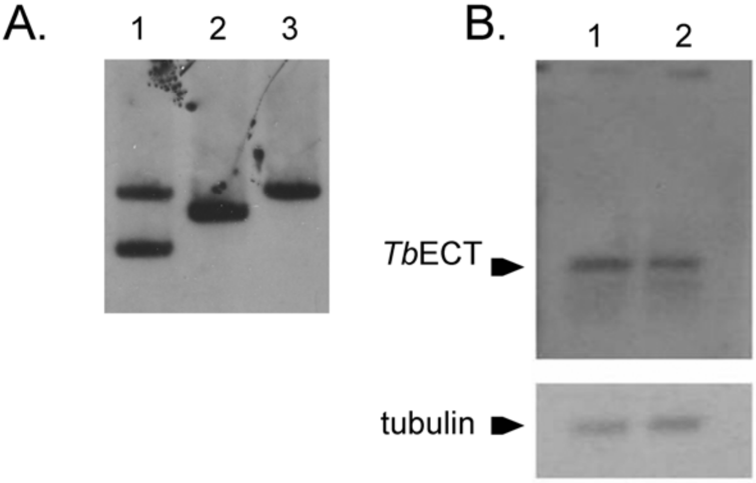

Figure S3. (A) Southern blot analysis. gDNA digested with *Xho*I (lane 1), *Bsa*I(lane 2) and *Nco*I (lane 3) (B) Northern blot analysis. Total mRNAs insect form (lane 1) and bloodstream form (lane 2) were separated on a gel, blotted and hybridized with *TbECT* [32P]-labelled probe (upper panel), and after stripping, reprobed with a [^{32}P]-labelled β-tubulin probe as loading control.

Figure S4

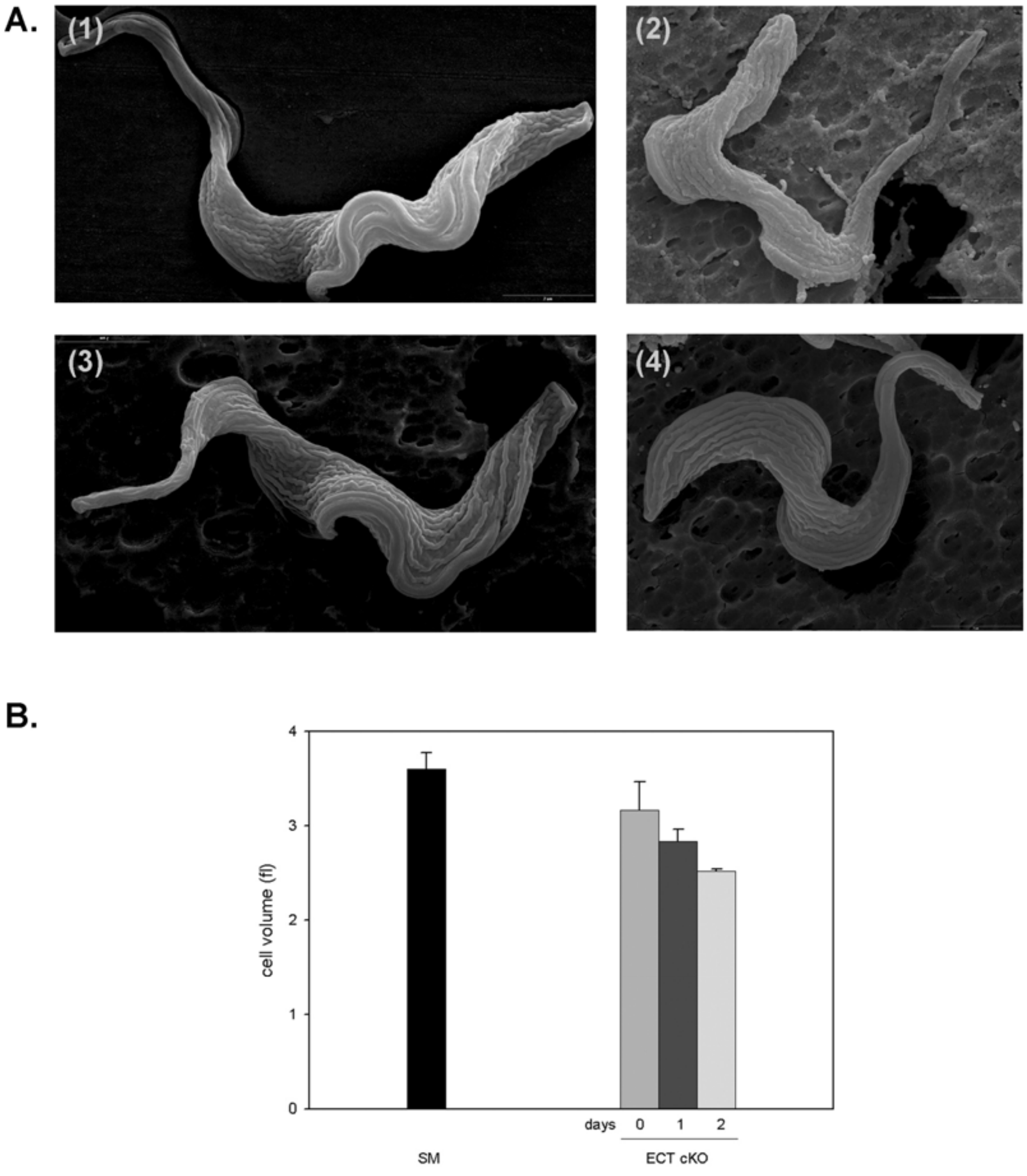

Figure S4. *Tb*ECT deprivation affects cellular dimensions. **(A)** Scanning electron microscopy of **(1)** SM parental cells, **(2)** *TbECT* cKO cells grown in the presence of tetracycline, *TbECT* cKO cells grown in the absence of tetracycline for 36 hours **(3,4)**. Scale bars, 2 μm. **(B)** The average cell volume was assessed for SM parental cells (black bar) and for *TbECT* cKO cells at different time points after tetracycline removal from the media (0, 1 and 2 days). Every measurement was performed at least in triplicate.

Figure S5

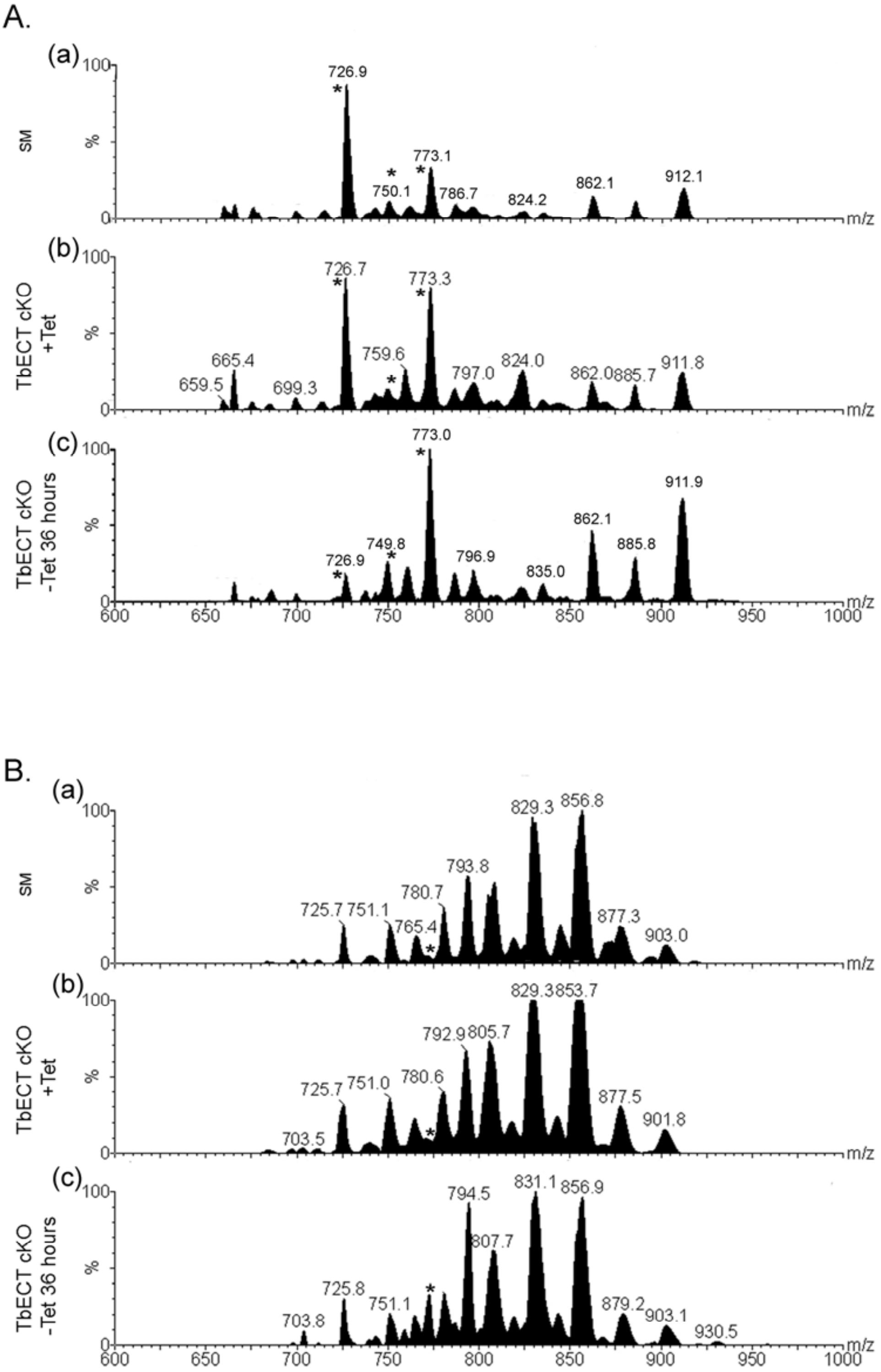

Figure S5. ESI-MS negative **(A)** and positive **(B)** ion survey scan spectra of total lipid extract from *T. brucei* bloodstream-form cells. **(a)** SM parental cells; **(b)** *TbECT* cKO cells grown in the presence of tetracycline; **(c)** *TbECT* cKO cells grown in the absence of tetracycline for 36 hours. Asterisks highlight relevant peaks described in the text.

Figure S6

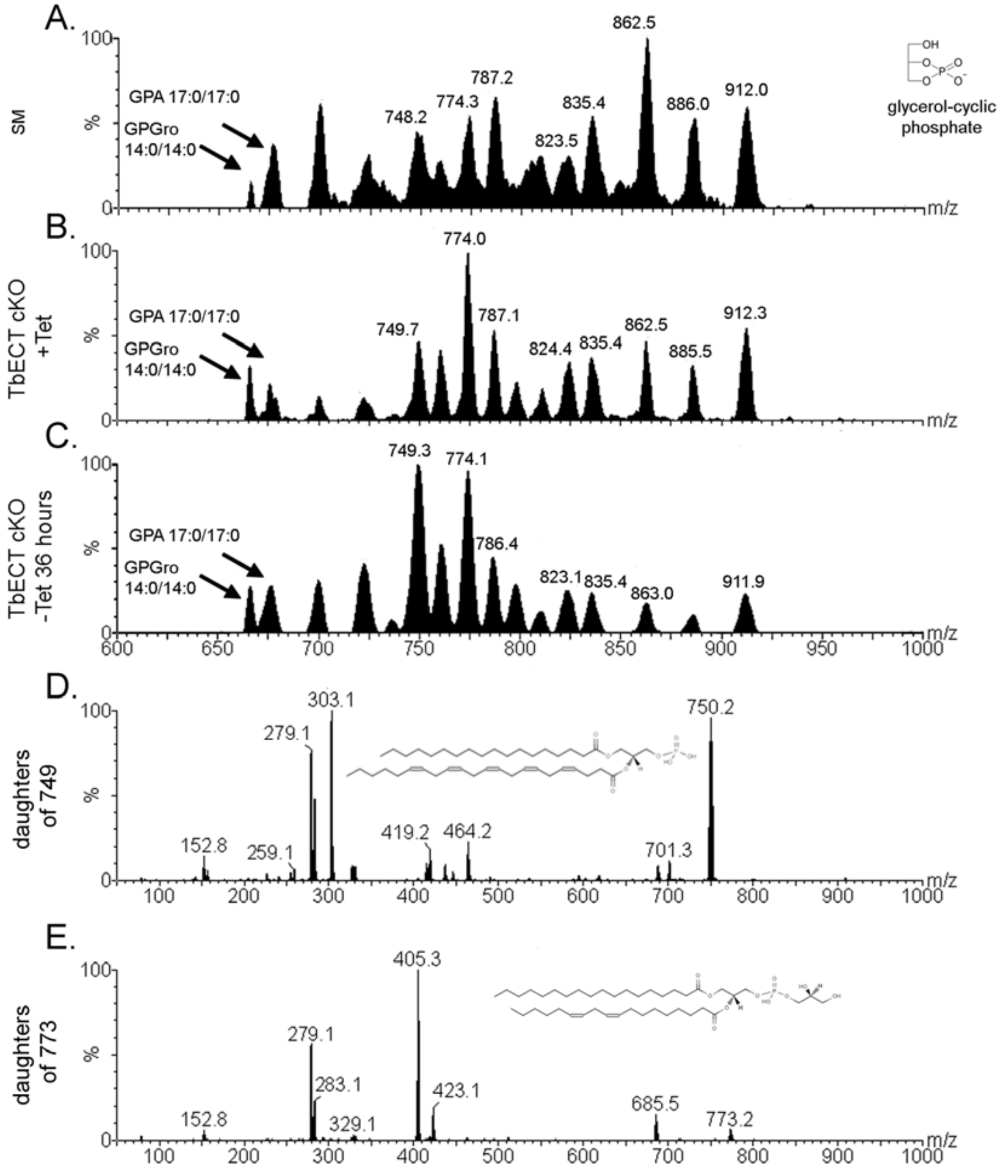

Figure S6 (A-C) Detection of glycerophospholipids by ESI-MS/MS by precursor ion scanning in negative ion mode for m/z 153 (glycerol-cyclic phosphate). The arrows indicate the peaks of internal standards GPGro (14:0/14:0) and GPA (17:0/17:0). **(A)** SM parental cells; **(B)** *TbECT* cKO cells grown in the presence of tetracycline; **(C)** *TbECT* cKO cells grown in the absence of tetracycline for 36 hours. **(D)** Daughter ion spectrum of the m/z 749 [M-H]$^-$ ion. **(E)** Daughter ion spectrum of the m/z 773 [M-H]$^-$ ion.

Figure S7

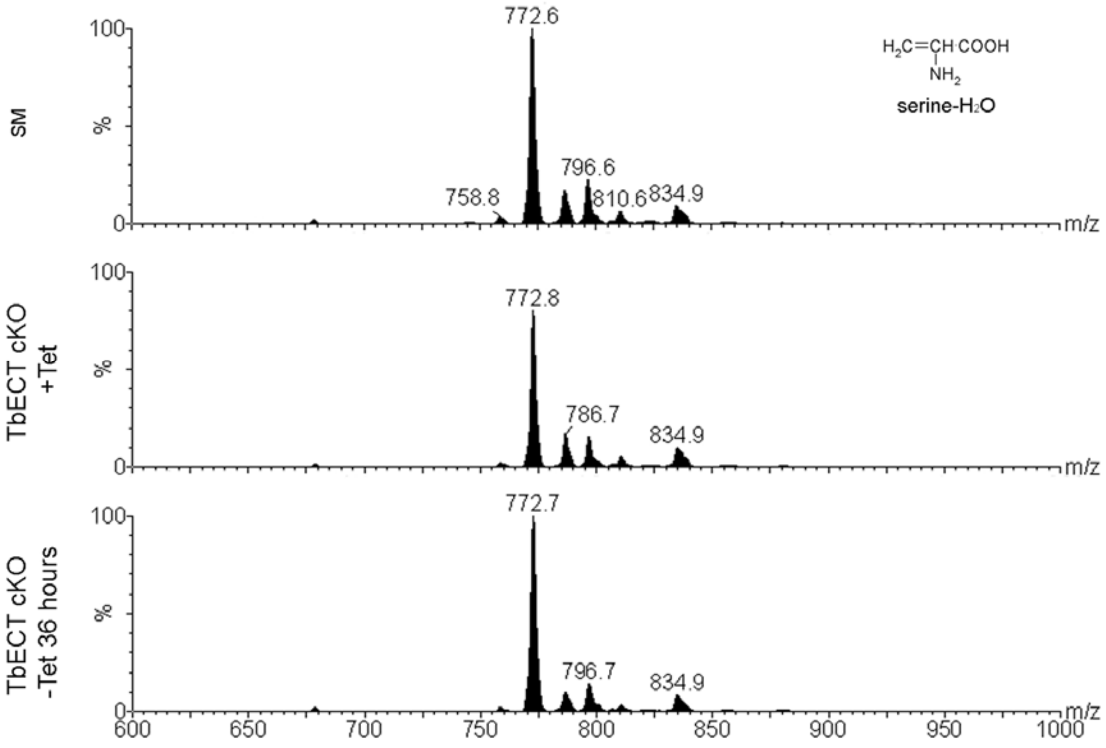

Figure S7. Serine-containing phospholipids were analysed by ESI-MS/MS in negative ion mode by neutral loss of the collision induced fragment serine – H_2O at m/z 87. Upper panel, SM parental cells; middle panel, *TbECT* cKO cells grown in the presence of tetracycline; lower panel, *TbECT* cKO cells grown in the absence of tetracycline for 36 hours.

Figure S8

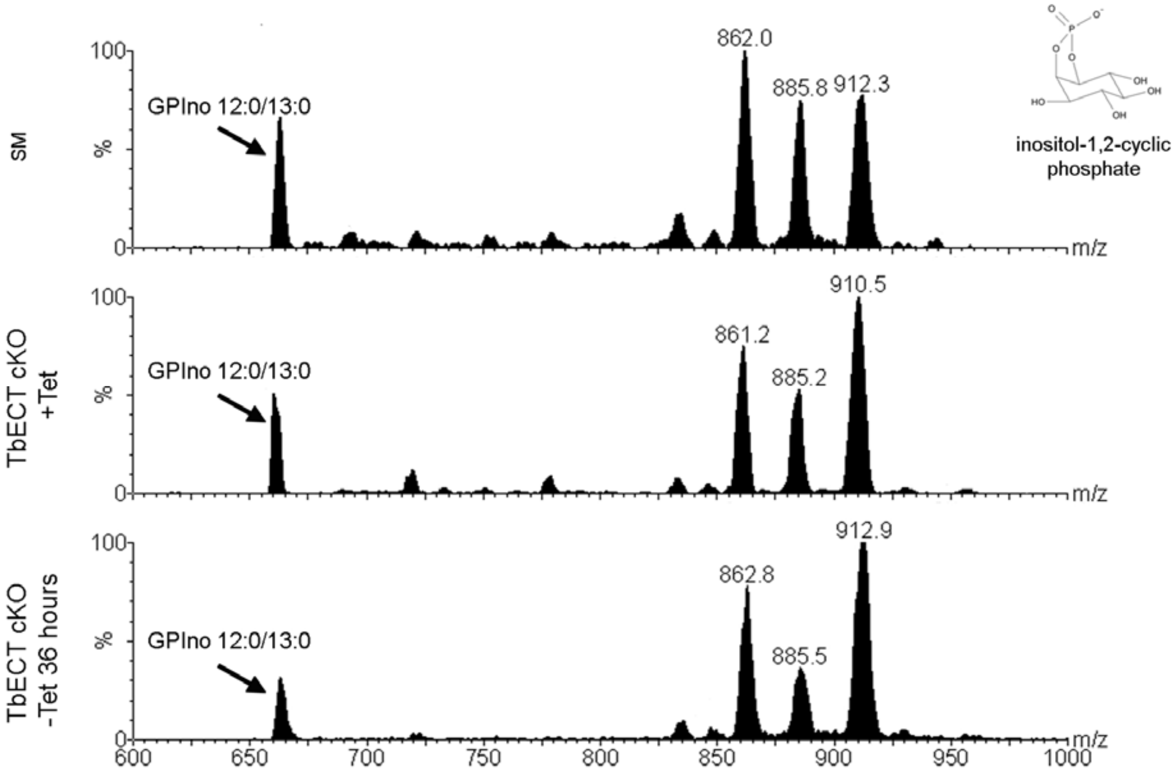

Figure S8. Inositol-containing phospholipids were analysed by ESI-MS/MS in negative ion mode using parent-ion scanning of the collision induced fragment inositol-1,2-cyclic phosphate at m/z 241. The arrows indicate the peaks of internal standards GPIno (12:0/13:0). Upper panel, SM parental cells; middle panel, *TbECT* cKO cells grown in the presence of tetracycline; lower panel, *TbECT* cKO cells grown in the absence of tetracycline for 36 hours.

Table S1: Mass spec analysis of GPEtn molecular species in bloodstream *T.brucei*

m/z[a]	*HR m/z*[b]	Lipid[c]	Principal component[d]	*Theor m/z*[e]
696	696.50	a-34:4	e-16:1/18:3	696.4973
**698**	**698.51**	**a-34:3**	**e-16:1/18:2**	**698.5130**
700	700.53	a-34:2	a-16:0/18:2	700.5286
702	702.55	a-34:1	a-16:0/18:1	702.5443
710	710.48	34:4	16:1/18:3	710.4766
**712**	**712.50**	**34:3**	**16:1/18:2**	**712.4923**
714	714.51	34:2	16:0/18:2	714.5079
716	716.53	34:1	16:0/18:1	716.5236
718	718.56	34:0	16:0/18:0	718.5392
724	724.53	a-36:4	e-18:1/18:3	724.5286
**726**	**726.54**	**a-36:3**	**e-18:1/18:2**	**726.5443**
**728**	**728.56**	**a-36:2**	**a-18:1/18:1**	**728.5599**
730	730.58	a-36:1	a-18:0/18:1	730.5756
732	732.59	a-36:0	a-18:0/18:0	732.5912
738	738.51	36:4	18:2/18:2	738.5079
740	740.53	36:3	18:1/18:2	740.5236
742	742.54	36:2	18:0/18:2	742.5392
**744**	**744.56**	**36:1**	**18:0/18:1**	**744.5549**
746	746.57	36:0	18:0/18:0	746.5705
748	748.54	a-38:6	e-18:1/20:5	748.5286
750	750.55	a-38:5	e-18:1/20:4	750.5443
752	752.57	a-38:4	a-18:0/20:4	752.5599
**754**	**754.58**	**a-38:3**	**a-18:0/20:3**	**754.5756**
756	756.60	a-38:2	a-18:0/20:3	756.5912
758	758.62	a-38:1	a-18:0/20:2	758.6069
760	760.62	a-38:0	a-18:0/20:1	760.6225
762	762.51	38:6	18:2/20:4	762.5079
764	764.52	38:5	18:1/20:4	764.5236
766	**766.54**	**38:4**	**18:0/20:4**	**766.5392**
768	768.56	38:3	18:0/20:3	768.5549
770	770.57	38:2	18:0/20:2	770.5705
772	772.59	38:1	18:0/20:1	772.5862
**774**	**774.55**	**38:0 or a-40:7**	**e-18:1/22:6**	**774.5443**
776	776.57	a-40:6	a-18:1/22:5	776.5599
788	788.54	40:7	18:2/22:5	788.5236
790	790.55	40:6	18:1/22:5	790.5392
**792**	**792.55**	**40:5**	**18:1/22:4**	**792.5549**
794	794.57	40:4	18:0/22:4	794.5705
796	796.59	40:3	18:0/22:3	796.5862
820	820.59	42:5	20:0/22:5	820.5862
822	822.60	42:4	20:0/22:4	822.6018
**824**	**824.63**	**42:3**	**20:0/22:3**	**824.6175**
826	826.64	42:2	20:0/22:2	826.6331
828	828.67	42:1	20:0/22:1	828.6488

[a] $[M-H]^-$ ions over charge.
[b] $HR[M-H]^-$ ions over charge (high resolution survey scan) ±0.05m/z
[c] Peak identities refer to total number of carbon atoms and double bonds.
[d] Precise fatty acyl constituents were deduced from daughter ion scanning, and when results were ambiguous, the literature was consulted to determine, where possible, the most likely candidate.
[e] All of the molecular species detected within the area of the peak are contained within the lowest and highest mass range outliers listed, the degree of unsaturation decreases by one from the lowest mass in each series until the highest mass is met. e = plasmenyl (alkenylacyl); a = plasmanyl (alkylacyl).

Table S2: Substrate specificity of *Tb*ECT

Compound	*Tb*ECT Relative Rate[a] (SD)
CTP	100.0 (6.8)
dCTP	31.6 (9.1)
CDP	0.0 (13.0)
AMP	3.1 (6.2)
ADP	3.1 (1.2)
ATP	3.1 (5.8)
UTP	7.0 (2.9)
GTP	6.1 (0.47)

[a] The various nucleotides were added to the reaction mixture in place of CTP at the constant concentration of 100 μM. The reaction rate was compared with that elicited by CTP at a fixed, saturating concentration of Etn-P to give a relative rate (CTP = 100).
